# Post-Synthetic Enzymatic and Chemical Modifications for Novel Sustainable Polyesters

**DOI:** 10.3389/fbioe.2021.817023

**Published:** 2022-01-05

**Authors:** Fady Abd El-malek, Alexander Steinbüchel

**Affiliations:** International Center for Research on Innovative Biobased Materials (ICRI-BioM)—International Research Agenda, Lodz University of Technology, Lodz, Poland

**Keywords:** post-synthetic modification, biopolymers, polyhydroxyalkanoates, lipases, chemical modification, enzyme modification

## Abstract

Because of their biodegradability, compostability, compatibility and flexible structures, biodegradable polymers such as polyhydroxyalkanoates (PHA) are an important class of biopolymers with various industrial and biological uses. PHAs are thermoplastic polyesters with a limited processability due to their low heat resistance. Furthermore, due to their high crystallinity, some PHAs are stiff and brittle. These features result sometimes in very poor mechanical characteristics with low extension at break values which limit the application range of some natural PHAs. Several *in vivo* approaches for PHA copolymer modifications range from polymer production to enhance PHA-based material performance after synthesis. The methods for enzymatic and chemical polymer modifications are aiming at modifying the structures of the polyesters and thereby their characteristics while retaining the biodegradability. This survey illustrates the efficient use of enzymes and chemicals in post-synthetic PHA modifications, offering insights on these green techniques for modifying and improving polymer performance. Important studies in this sector will be reviewed, as well as chances and obstacles for their stability and hyper-production.

## Introduction

PHAs are biodegradable polyesters with a wide range of building blocks. PHAs became quite attractive, as they have similar thermal and material properties like conventional thermoplastics (Abd El-malek et al., 2020; Abd El-malek et al., 2021). Bacterial PHAs are produced from a variety of renewable feedstock and by biological processes such as beer brewery waste, food waste, algal biomass, rice mill effluent, waste frying oil, etc., mostly through fermentation (El-Malek et al., 2021). Different types of PHAs, including homopolymers such as polyhydroxybutyrate (PHB) and copolymers like 3-hydroxybutyrate-*co*-3-Hydroxyvalerate, have been produced using both, the wildtype of *Ralstonia eutropha* and others as well as various recombinant microorganisms (Chung et al., 2011; Gumel et al., 2012b). Bacteria accumulate these polyesters as insoluble intracellular granules when they are cultivated under imbalanced conditions including excess carbon and limitation in nitrogen and phosphate (El-Malek et al., 2020). Many carbon sources, such as alcohols, alkenes, alkanes, fatty acids and sugars can be utilized as feedstock, allowing the production of a wide range of polyesters and monomer units. The constituent of the polymers vary and is highly influenced by the substrates provided, growth conditions, and bacterial metabolism, specially enzymatic behaviors (Bassas-Galià et al., 2015).

**FIGURE 1:**
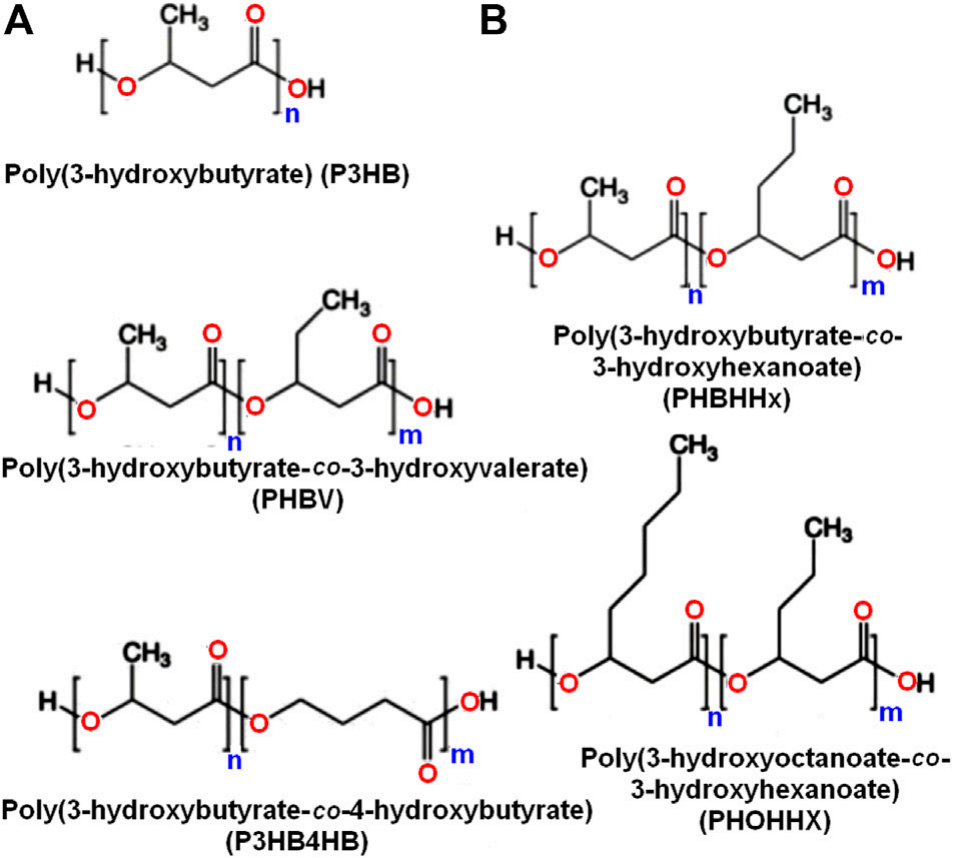
A chemical structure illustration showing scl and mcl-PHA polyesters with short side chain **(A)** and others with long side chain **(B)**.

These polymers may generally be categorized as short chain length (scl) PHA and medium chain length (mcl) PHA according to the number of carbon atoms in the single unit (Figure 1). Scl-PHAs, like PHB, are crystalline and have a polypropylene-like tensile strength (40 MPa), but are more fragile. Alternatively, mcl-PHAs possess low *T*_*m*_ (<50°C), low tensile strength and are amorphous or semi-crystalline elastomers. Over the last decades, the manufacturing of PHB and mcl-PHA copolymers has expanded widely (Steinbüchel and Valentin, 1995; Reddy et al., 2003). However, the utilization of PHAs is limited in many applications because of their hydrophobicity and the absence of chemical functionalities that depend on the constituents of PHAs (Kai and Loh, 2014; Gumel et al., 2015). Post-synthetic modifications including the materials such as coating (Wang et al., 2003), blending (Yang et al., 2002), plasma treatment (Tezcaner et al., 2003) and electro-spinning (Kwon et al., 2007), modifications of the polymer molecules by *in vivo* and *in vitro* enzyme treatment (Gumel et al., 2015) or chemical modification like carboxylation (Lee and Park, 2000; Renard et al., 2007), epoxidation (Park et al., 1998b; Sparks and Scholz, 2008) and hydroxylation (Sparks and Scholz, 2008; Kai and Loh, 2014) can be introduced to broaden their usefulness.

PHA synthases are important enzymes in PHA biosynthesis, they catalyze the polymerization of (*R*)-hydroxyalkanoyl moieties in (*R*)-hydroxyalkanoyl-coenzyme A (HACoA), resulting in the simultaneous release of CoA (Rehm, 2003; Taguchi and Doi, 2004). The HACoA supply pathway to PHA synthases also influences monomer compositions, which are heavily influenced by the cell metabolic pathways such as *de novo* fatty acid production, catabolic pathways and substrate feedings. PHAs containing the desired functional groups are not synthesised naturally by the bacterial PHA synthases; such polyesters need functional groups that are provided in the carbon sources used for the process of microbial fermentation harboring the required functions (Rehm, 2003).

The majority of commercial lipases have been employed to accelerate the formation of ester linkages in micro-aqueous environments (Gumel et al., 2012a). Most of these lipases are commercially available phospholipase, which exhibit hydrolytic activity (Mishra et al., 2009). On the other hand, the potential of these enzymes for the esterification of PHAs in micro-aqueous catalysis has yet to be discussed. For example, Lecitase TM Ultra (EC 3.1.1.32) is an engineered phospholipase A1 produced by *Thermomyces lanuginosus* with both lipase activity and the engineered stable phosphatase enzyme.

Sugars have been used in the chemo-enzymatic functionalization of polymers such as polyvinyl (Tokiwa et al., 2000), polyacrylate (Martin et al., 1992), and poly (ε-caprolactone) (PCL) (Chen et al., 1995). However, these polymers were produced with di-carboxylic acid spacer arms, which may have contributed to the polymer’s increased hydrophobicity rather than to its hydrophilicity. In several cases, the functionalized polymers produced by this method contain harmful chemical contaminants, making it unsuitable for biomedical applications. Furthermore, the enzymatic functionalization of bacterial PHA with carbohydrates is regiospecific. In addition, the used solvent may impact on single-step enzymatic functionalization of mcl-PHA. Different organic solvents, including dichloromethane, chloroform, isooctane, and dimethyl sulfoxide (DMSO), were tested for their effects on the Lecitase TM Ultra, which catalyzes the functionalization of mcl-PHA with glucose moieties. As compared to non-functionalized PHA, the molecular weight analysis of the functionalized polymer indicated a general reduction of the average molecular weight (Mw 15.2 ± 0.3 kDa) with a rising number-averaged molecular weight (Mn) of 10.0 ± 0.5 kDa and a polydispersity index (PDI) of 1.5, while the values were (Mw 55.7 kDa, Mn 13.6 kDa and PDI 4.1) for the non-functionalized polymer. This observation was confirmed further by the thermal decomposition temperature (*T*_*d*_) values of 298.9°C for non-functionalized mcl-PHA and 306.4°C for functionalized mcl-PHA. Furthermore, the requirement for chemo-synthetic stages was avoided by employing H_2_O_2_ as a micro-initiator to efficiently manufacture the carbohydrate polymer utilizing the enzyme (Gumel et al., 2013a).

**FIGURE 2:**
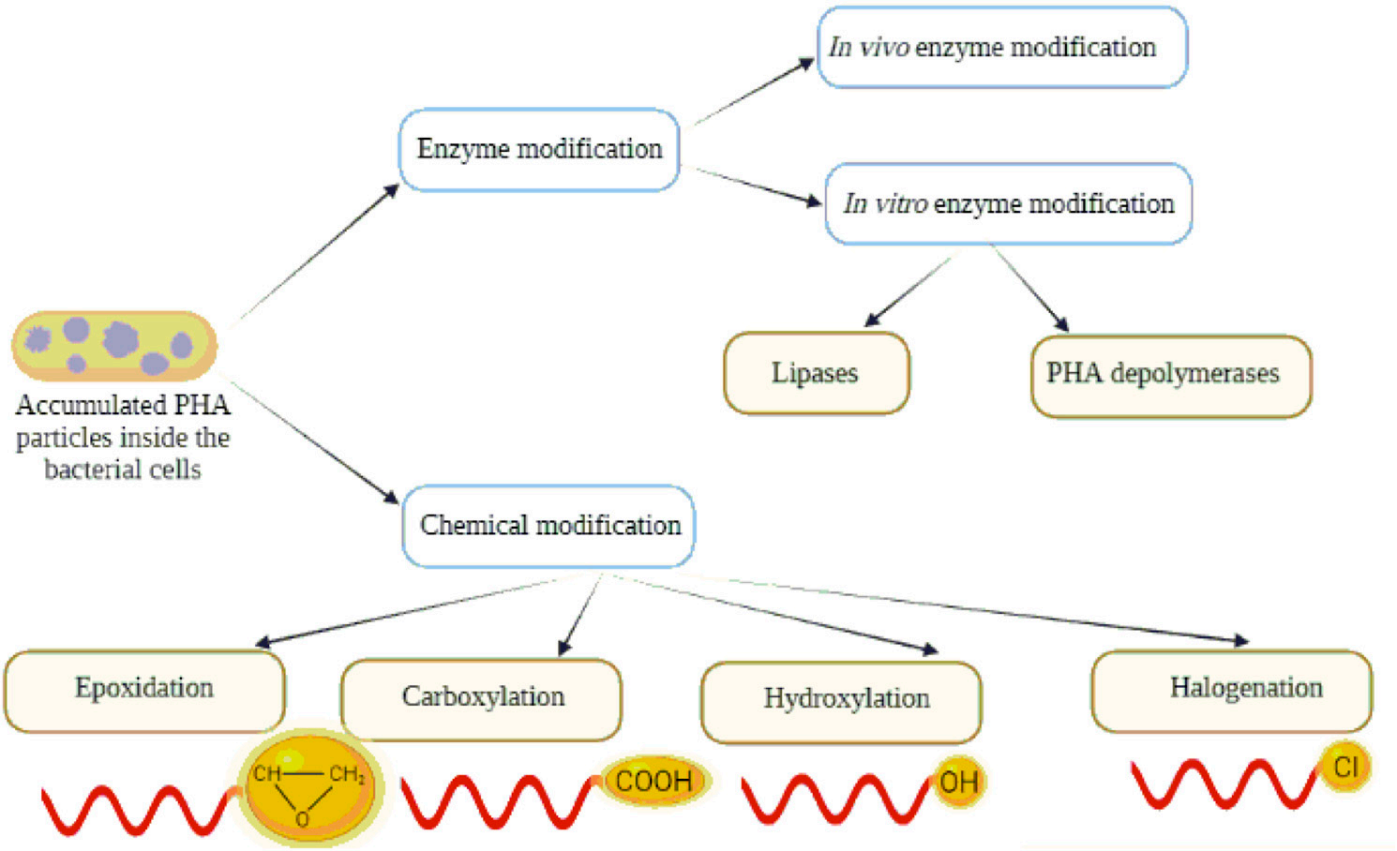
An illustration showing all possible enzymatic and chemical modifications of PHAs.

We focused our efforts for alternate methods on the generation of new PHAs by polymer modification to extend the spectrum of PHAs applications. This review article shades light on how enzymatic and chemical PHA modifications (Figure 2) can affect the sustainability of the polymer. Moreover, it describes the applicability of the modified polymers in several fields.

## Enzymatic Modifications of Polyhydroxyalkanoates

Enzyme-mediated PHA modification processes are thought to be a selective, gentle and ecologically acceptable approach. This section discusses PHA modifications by enzymatic degradation and/or synthesis techniques in both *in vitro* and *in vivo* processes. The enzymatic modification of PHA by utilizing PHA degradation products is also included in the discussion.

### *In Vitro* Enzymatic Modification

Enzymes catalyze numerous processes that would be difficult to be done with chemical catalysts and are critical in the synthesis and modification of various polymers. Changes in the reaction media can have a considerable impact on the polymeric structure, yield, polydispersity and molecular weight. An understanding of the various enzyme reaction mechanisms is required to modify the polyesters for industrial applications.

#### Lipases

Lipases are considered as a subclass of esterase enzymes that can hydrolyze lipids, fats and esters in aqueous media systems; therefore, they are classified as hydrolases belonging to the EC3.1.1.3 class. Lipases are one of the most widely used enzymes class in industry. They are among the most diverse enzymes since they may catalyze a wide range of reactions including alcoholysis, aminolysis, epoxidation, esterification, inter-esterification, hydrolysis and peroxidation (Kawata and Ogino, 2009). The capacity of lipases to catalyze this wide range of reactions allows them to find uses in biodiesel production, food modification, pharmaceuticals and medications manufacture. These enzymes can be microbially produced; a thermostable *Bacillus subtilis* lipase was previously developed by Kamal et al. (Kamal et al., 2011; Kamal et al., 2013).

Most lipases contain amino acid sequences that are similar, particularly within the catalytic domain, His-X-Y-Gly-Z-Ser-W-Gly or Y-Gly-His-Ser-W-Gly, where W, X, Y, and Z are unidentified amino acid residues. These enzymes are specific for ester bonds (Lipases catalyze the hydrolysis of ester bonds), without the occurrence of unwanted byproducts (Santaniello et al., 1993). Lipases have been frequently used as biocatalyst in industry due to their flexible pH range, excellent thermal stability and their ability to be reused if immobilized (Yadav and Devi, 2004). Yadav and Devi (Yadav and Devi, 2004) conducted a comprehensive study of the esterification and transesterification of tetrahydrofurfuryl alcohol, taking into account the influence of numerous factors. Novozym 435 was identified as the most effective catalyst among all immobilized lipases. Transesterification was also shown to be inhibited by both substrates and products, suggesting that esterification is preferable to transesterification (Yadav and Devi, 2004).

For the lipase catalyzed modifications of PHAs; Mukai et al. (Mukai et al., 1993) showed that lipases obtained from eukaryotes exhibit broad specificities, with the ability to erode poly (3-hydroxypropionate) [P (3HP)], poly (4-hydroxybutyrate) [P (4HB)], poly (5-hydroxyvalerate) P (5HV), and poly (6-hydroxyhexanoate)P (6HH) films when compared to prokaryote lipases, which could hardly degrade all polyesters except P (3HP). This demonstrates that lipases from prokaryotes have high substrate specificities for PHA hydrolysis. Enzymatic degradation of PHA films in the presence of microbial PHA depolymerases was also performed for 5 h at 37°C and pH 7.4. In terms of enzymatic assaults on P [(*R*)-3HB] films, PHA depolymerases differ from microbial lipases. Lipases in excess did not cause the P [(*R*)-3HB] film to erode. Furthermore, lipases did not digest P [(*R*)-3HB] granules with a higher surface area (Mukai et al., 1993). Research utilizing PHA depolymerases, which are similarly generated by microorganisms, revealed the degradation behavior of PHAs. The catalytic domain of these PHA depolymerases is defined by the catalytic triad S-H-D (serine, histidine, and aspartate residues), with the serine positioned in the typical lipase-box pentapeptide Gly-X1-Ser-X2-Gly (Urbanek et al., 2020). As a result, the conformational properties of this LID region should be crucial for enzymatic activity.

According to Jaeger et al. (Jaeger et al., 1995), *B. subtilis*, *Burkholderia lemoignei*, *Pseudomonas aeruginosa*, *P. alcaligenes*, and *P. fluorescens* lipases prefer ɷ-hydroxyalkanoic acid to α-hydroxyalkanoic acid. Because there are no alkyl side chains in the polymer backbone, the main polymeric chain is more flexible and hydrophilic, resulting in improved interaction between the polyester chain and the active site of lipases (Ch’ng and Sudesh, 2013). The capability of bacterial lipases to degrade PHAs is noteworthy for two reasons; 1) It demonstrates the extraordinary flexibility of lipases in terms of hydrolysis of various substrates, and 2) the growing economic potential of different PHA, which is primarily based on their biodegradability, may be bolstered by the fact that not only specialized PHA depolymerases, but also lipases occurring in numerous bacteria, might contribute to the biodegradation of these polymers (Jaeger et al., 1995). In addition, Ch’ng and Sudesh established a unique technique for sensitive detection and measurement of triglyceride lipase PHA degrading capacity. Densitometric studies revealed that 12 of 14 lipases from fungal, bacterial, and animal origins could degrade P (3HB-*co*-92 mol% 4HB) thin film. When the specific activities of lipases from eukaryotic origins were examined using PHA, p-nitrophenyl laurate (pNPL), and olive oil as substrates, three lipases from eukaryotic sources exhibited greater preferences on the polymer film. Furthermore, bacterial lipases were shown to have a substantial capacity to digest PHA (Ch’ng and Sudesh, 2013).

Different variables necessitate particular consideration for lipase-catalyzed degradation and esterification processes. The temperature of the reaction is important for enzyme stability and the solubility of substrates such as sugar moieties and alcohol in order to optimize lipase catalyzed esterification activity in organic solvents because the main problem with hydrophilic moieties is that they are insoluble in organic solvents (Ha et al., 2010). It has been reported that a high yield of poly (10-O-3-hydroxyacyl-sucrose) was produced using Novozyme 435 at a temperature of 50°C. This was demonstrated to be related to an improvement in substrate dissolution (Gumel et al., 2013a). The reaction rate for lipase catalyzed PHA degradation in toluene was about the same at 40 and 60°C (Pastorino et al., 2004). However, the rate of PHA degradation by lipases has been shown to be influenced by parameters such as lipase concentration (Ch’ng and Sudesh, 2013), lipase type (Jaeger et al., 1995) and degree of initial crystallinity.

Snoch et al. (Snoch et al., 2019) recently presented the development of a biocatalytic synthesis method for the preparation of unique glucose esters based on PHA derived monomers, namely mixtures of (*R*)-3-hydroxy-3-phenylpropionic acids and (*R*)-3-hydroxy-5-phenylpentanoic or (*R*)-3-hydroxyheptanoic acids and (*R*)-3-hydroxynonanoic arising from two types of PHA sources produced by bacterial fermentation of *P. putida* CA-3 on 5-phenylvaleric or nonanoic acids, respectively. The functionalization of PHA derived acids was performed by addition of a 2,2,2-trifluoroethyl trifluoromethyl sulphate moiety. In water-free organic solvent environments, the virgin mixtures or their modified equivalents were linked to glucose catalyzed by a lipase. The resultant new sugar fatty acids esters (SFAEs) were purified and characterized (Snoch et al., 2019).

Recently, Bhatia et al. utilized modified *E. coli* YJ101 to produce P (3HB-*co*-3HV) copolymer which was subsequently functionalized with ascorbic acid using *Candida antarctica* lipase B-mediated esterification. The copolymer P (3HB-*co*-3HV)-ascorbic acid had a lower degree of crystallinity (9.96%), a higher thermal degradation temperature (295°C), and a higher hydrophilicity (68°) in comparison to P (3HB-*co*-3HV). Furthermore, the P (3HB-*co*-3HV)-ascorbic acid biomaterial had a 14 %scavenging effect on 1,1-diphenyl-2-picryl-hydrazyl (DPPH) and a 1.6 fold-improvement in biodegradability. By adding functional groups to the polymer, it could be an excellent way to improve their biodegradability, economic value and crucial medical uses (Bhatia et al., 2019).

#### Extracellular Polyhydroxyalkanoates Depolymerases

**FIGURE 3:**
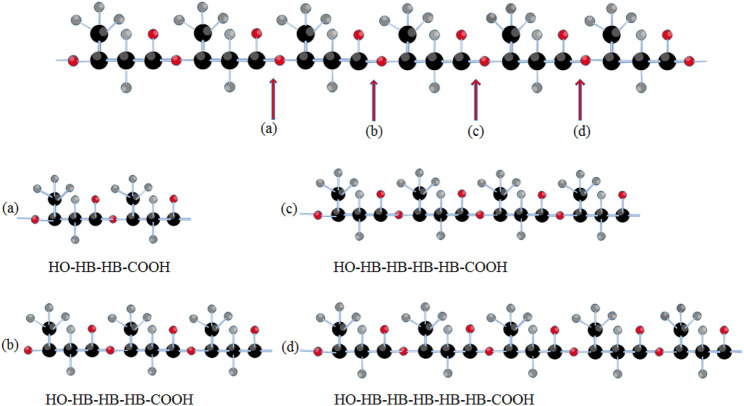
An illustration for bond cleavage in PHBH polymeric chain during the PHB depolymerase action producing different oligomers.

Several PHA-degrading bacteria have been identified, and their capacity to produce extracellular PHA depolymerases might be used to their advantage. The degraded monomer can be isolated and utilized as a primer in subsequent modifications (Jendrossek and Handrick, 2002). Extracellular PHA depolymerases for mcl-PHA have been identified mostly in Gram-negative bacteria, such as *Alcaligenes faecalis* T1, while scl-PHA depolymerases have been investigated in *Streptomyces roseplus* SL3 and *P. fluorescens* GK13 (Gangoiti et al., 2012). The *A. faecalis* T1 PHA depolymerase is a serine hydrolase enzyme with the lipase box consensus sequence Gly-X-Ser-X-Gly, and it is highly hydrophobic. PHA depolymerase’s catalytic triad includes a serine residue that works as a nucleophile with aspartate (or glutamate) and histidine to stabilize it (serine-histidine-aspartate) (Knoll et al., 2009; Jaeger et al., 1995). Moreover, It was found that PHB depolymerases from *R. pickettii* T1 can degrade poly (3-hydroxybutyrate-*co*-3-hydroxyhexanoate) (PHBH) into a mixture of the monomers and oligomers such as (3-hydroxybutyrate-3-hydroxyhexanoate) 3HB–3HH dimer, and 3HB– 3HB–3HH trimer (Jeepery et al., 2021) (Figure 3). In addition, PHA depolymerases favors PHA with alkyl side chains (Jaeger et al., 1995).

PHA depolymerases are highly selective for chiral monomers, particularly for PHAs with alkyl side chains such as 3-hydroxybutyrate (3HB) rather than 4-hydroxybutyrate (4HB). The rate of enzymatic hydrolysis is largely determined by the composition of the polyester and the length of the PHA side chain (Mukai et al., 1993). Longer side chains provide steric barriers for the enzyme to be efficiently adsorbed on the polymer backbone chain.

The substrate concentration, stability, activity and temperature of enzymes are all important variables in enzymatic catalysis. The optimization of these variables will direct the enzyme’s activity in the desired direction. The ionizable groups in the active site of the enzyme were shown to be affected by the pH of the media. Therefore, the activity of poly (3-hydroxyoctanoic acid) [P (3HO)] depolymerase activity was shown to be highest at pH 9.5 and 40°C (Gangoiti et al., 2010).

The extracellular mcl-PHA depolymerase of *P. fluorescens* GK13 catalyzes P (3HO) hydrolysis. Thus, the extracellular P (3HO) depolymerase found in the culture broth of *P. fluorescens* GK13 cells cultivated in mineral media supplemented with P (3HO) as sole carbon and energy source was firmly adsorbed with high yield (Gangoiti et al., 2010). The addition of detergents and organic solvents increased the activity of the purified enzyme, and was maintained after treatment with an SDS-denaturing cocktail under both reducing and non-reducing conditions. The dimeric ester of 3-HO [(*R*)-3-HO-HO] was produced as the major product of the soluble enzyme after 24 h of hydrolysis. However, under the same circumstances, the immobilized enzyme catalyzes nearly full hydrolysis of the P (3HO) polymer to (*R*)-3-HO monomers (Gangoiti et al., 2010).

### *In vivo* Enzymatic Degradation of Polyhydroxyalkanoate

PHA granules accumulate inside the bacterial cells during the fermentation process under imbalanced cultivation conditions, i.e. in the presence of excess carbon source(s) and if essential nutrients such as phosphorous, sulfur, oxygen, nitrogen or potassium are not sufficiently available (Rofeal et al., 2021). If the microbes are starved of a carbon source in presence of abundant other nutrients, they start to produce intracellular PHA depolymerases and dimer hydrolases to degrade the accumulated PHA and continue to grow (Müller and Seebach, 1993). The monomers formed by intracellular degradation are then oxidized by the cells to generate acetoacetate. Lee et al. (Lee S. Y. et al., 1999) suggested a technique that includes continual restriction of the carbon supply and nutrient(s) in an anaerobic state in an attempt to bypass accumulated PHA hydrolysis. A lack of oxygen prevents cells from metabolizing the 3HB monomers derived from intracellular PHB degradation by reducing the concentration of R-3-hydroxybutyrate dehydrogenase thereby reducing the conversion of 3HB to acetoacetate (Lee S. Y. et al., 1999). Moreover, by *in vivo* depolymerization of intracellular PHAs, a new and effective technique for producing enantiomerically pure (*R*)-(-)-hydroxycarboxylic acids was devised. Several model compounds, including (*R*)-(-)-3-hydroxyalkanoic acids with 4–12 carbon atoms and (*R*)-(-)-3-hydroxy-5-phenylvaleric acid, may be produced by this technique. This approach might be also used to efficiently manufacture (*R*)-(-)-3-hydroxybutyric acid (Lee S. Y. et al., 1999).

A novel and efficient method for the production of enantiomerically pure (R)-(-)-hydroxycarboxylic acids by *in vivo* depolymerization of microbial polyester poly-hydroxyalkanoates (PHAs) was developed. Using this method, several model compounds, (R)-(-)-3-hydroxyalkanoic acids, consisting of 4–12 carbon at-oms, and (R)-(-)-3-hydroxy-5-phenylvaleric acid, could be prepared. In particular, (R)-(-)-3-hydroxybutyric acid could be efficiently prepared by this method. By providing the environmental condition in which cells possess high activity of intracellular PHA depolymerase and low activity of (R)-(-)-3-hydroxybutyric acid dehydrogenase, (R)-(-)-3-hydroxybutyric acid could be produced with a yield of 96% in only 30 min by *in vivo* depolymerization of polyhydroxybutyrate (PHB) accumulated in Alcalig-enes latus.

It has been reported that a high yield of 3HB (96%) may be produced in *A. latus* within a relatively short time period (30 min) utilizing a fed-batch culture method with sucrose as carbon source. Cells containing stored PHB were collected and cultured at pH 4 and 37°C to create an environment in which the cells display high activity of internal PHA depolymerase and low activity of (*R*)-(–)-3-hydroxybutyric acid dehydrogenase. Substrate concentration and extracellular pH have also been identified as variables that contribute to *in vivo* depolymerization (Lee S. Y. et al., 1999). Ren et al. (Ren et al., 2005) has shown that the best pH range at the beginning of intracellular PHA depolymerization by *P. putida* was 8–11, and pH 11 after the monomers began to be released (Ren et al., 2005). At an initial pH of 11, PHA including 3-hydroxyoctanoic acid and 3-hydroxyhexanoic acid decomposed in 9 h with an efficiency of more than 90% (w/w), and the yield of the corresponding monomers was likewise more than 90%. Unsaturated monomers were efficiently generated from PHA containing 3-hydroxy-6-heptenoic acid, 3-hydroxy-8-nonenoic acid, and 3-hydroxy-10-undecenoic acid under the same circumstances. The monomers (e.g., 3-hydroxyoctanoic acid) were then separated and purified using reversed phase semipreparative liquid chromatography (Ren et al., 2005).

Anis et al. investigated the *in-vivo* depolymerization of PHAs accumulated in *P. putida* Bet001 after 48 h of batch culture with lauric acid as carbon source and under nitrogen limitating conditions. In 0.2 M Tris–HCl buffer, pH 9, at 30°C, the depolymerization was carried out for 48 h. Unlike Lee et al., who used *P. aeruginosa* PAO1 (DSM 1707), the *P. putida* Bet001 strain generated (*R*)-3-hydroxyoctanoic acid (R3HO), (*R*)-3-hydroxyhexanoic acid (R3HHx), (*R*)-3-hydroxydecanoic acid (R3HD), and (*R*)-3-hydroxydodecanoic acid (R3HDD), in which R3HD has the highest depolymerization yield. It is unclear if the difference in yield represents an affinity of the PHA depolymerases for R3HO, R3HHx, and R3HDD for cell metabolism or whether they have yet to be hydrolyzed from the granules and thereby reflect a channeling of R3HO, R3HHx, and R3HDD toward cell metabolism (Lee S. Y. et al., 1999; Anis et al., 2018).

In another study, several strains of *Halomonas* were used to develop a new approach for producing R3HBA *in vivo*. 15.2 g L^−1^ of (*R*)-3-hydroxybutyric acid (R3HBA) were produced under microaerobic conditions using *Halomonas* sp. KM-1 from 16.4 g L^−1^ of PHB accumulated under aerobic conditions using glycerol as the sole carbon source (Kawata et al., 2014).

## Polymeric Surface Modification

PHAs are helpful in tissue engineering because of their superior mechanical characteristics, biocompatibility and degradability. These polymers have the potential to be highly effective as tissue scaffolds for implantation. The smooth surface of a solvent-cast PHA scaffold, on the other hand, is a substantial impediment to cell adhesion in tissue regeneration processes. This justifies PHA’s enzymatically catalyzed surface erosion (Chen et al., 2002). To obtain copolyesters of PHB and PCL, PHB and PCL were transesterified in the liquid phase with stannous octoate as catalyst. The effects of reaction parameters on transesterification were studied, including reaction temperature, concentration of catalyst and time for reaction. Therefore, the study findings revealed that raising the reaction temperature as well as the reaction duration were beneficial for the transesterification. The block copolymers were produced *via* transesterification of PHB and PCL. The thermal properties of the copolyesters changed noticeably as the PCL concentration in the copolyesters increased. The insertion of PCL segments into PHB chains, on the other hand, had no effect on its crystalline structure. Furthermore, in comparison to pure PHB, the thermal stability of the copolyesters was marginally enhanced (Chen et al., 2002).

**FIGURE 4:**
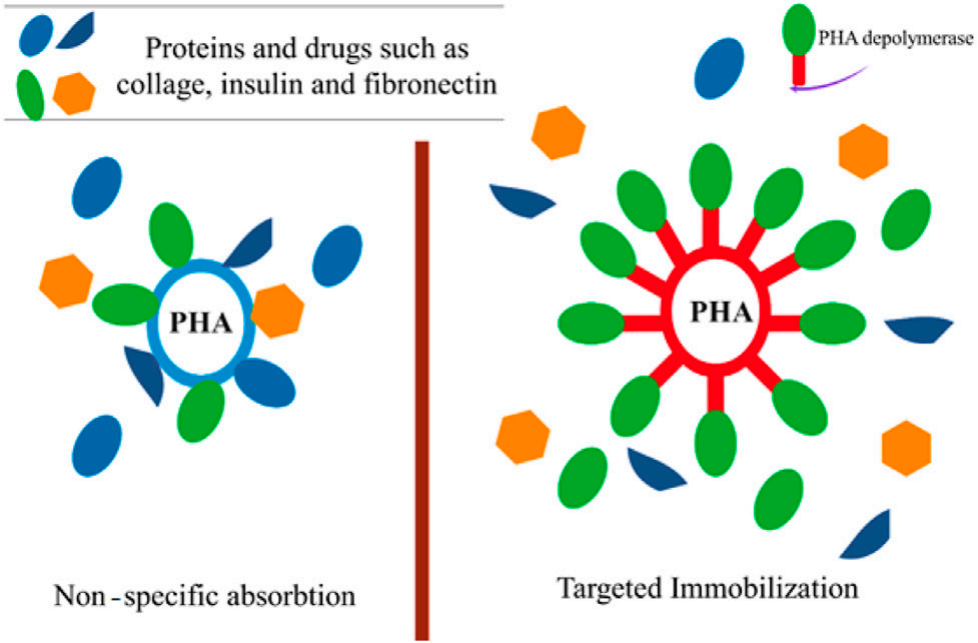
The usage of extracellular PHA depolymerase to immobilize specific proteins and drugs on the polyester surface.

The PHA surface is devoid of ligands that are capable of coupling with a bioactive molecule in targeting devices or biosensors. As a result, PHA surface roughening or erosion is required to create a corrugated material (wave-shape) to immobilize bioactive molecules such as collagen (Liu et al., 1993), insulin (Kim et al., 2000) and fibronectin (Ito et al., 1993), improving their cell adhesion or cell proliferation properties and thereby extending their biomedical uses. In the study of Ihssen et al. the extracellular PHA depolymerase produced by *P. fluorescens* was used as the capture ligand to immobilize a fusion protein on 200–300 nm mcl-PHA microbeads (Figure 4) (Ihssen et al., 2009).

These mcl-PHA microbeads might be used as a probe for protein targeting in protein purification and microarrays as well as for medication delivery. Furthermore, the binding capability was shown to be equivalent to similar-sized polystyrene particles frequently employed for antibody immobilization in clinical diagnostics (Ihssen et al., 2009). In addition, the use of such nanoparticles as drug delivery devices for targeted release to specific areas of the body has emerged as a potential therapy option for cancer (Shrivastav et al., 2013). Incorporating the tumor-specific ligand RGD4C with PHA synthase improves the adhesion of PHA nanoparticles to MDA-MB231 breast cancer cells (Lee et al., 2011). The excellent connection between the hydrophobic surface of the PHB nanoparticle and the PHB chain produced by the enzyme fused with a particular ligand, offered a straight forward method of functionalizing nanoparticles with active protein layers in an aqueous environment. PHB nanoparticles were loaded with the model drug molecule using an oil-in-water emulsion solvent evaporation technique, and the surface of the nanoparticles was functionalized with the tumor-specific ligand (RGD4C) coupled with PHA synthase, which triggered the coupling process. The functionalized PHB nanoparticles exhibited a particular affinity for MDA-MB 231 breast cancer cells, suggesting that the tumor-specific ligand RGD4C was successfully displayed on the surface of the PHB nanoparticles by enzymatic modification, conferring targeting capacity on the drug carrier (Figure 4) (Lee et al., 2011).

**FIGURE 5:**
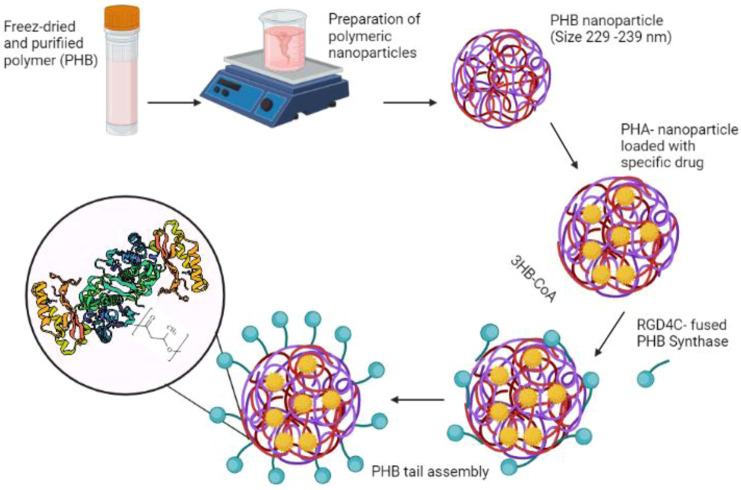
The functionalization of PHB nanoparticle’s surface with the tumor-specific ligand (RGD4C) coupled with PHA synthase.

PHA nanoparticles’ surface functionalization is required to increase drug delivery efficiency to the target cells. This can be accomplished by blocking PHA-protein copolymerization. PHA synthase is the key enzyme catalyzing the polymerization of the hydroxyacyl moieties of hydroxyacyl-Coenzyme A. PHA synthase and the polymer’s hydrophobic chain, on the other hand, were covalently bound during *in vitro* PHA synthesis, resulting in the formation of an amphiphilic block copolymer in which PHA synthase can be further modified through protein engineering to improve the protein-PHA copolymer (Kim et al., 2009). Moreover, Kim et al. (Kim et al., 2009) reported on modifications of PHA nanoparticles with average diameters of 233 ± 28 nm, 229 ± 14 nm, or 239 ± 21 nm utilizing the native, N-terminus fusion enzyme, and C-terminus fusion enzyme, respectively. This study compared three enzymes: one native and two engineered forms, while RGD4C was fused to the enzyme’s amino (N-terminus) or carboxy (C-terminus) terminus. The use of PHA synthase from *R. eutropha* H16 in aqueous solution at room temperature for the production of protein-PHB copolymer micelles (including polymerization and self-assembly of 3-hydroxybutyryl-CoA (3HB-CoA) resulted in the formation of the hydrated shell. Concurrently, hydrophobic drug molecules or other functional agents might be introduced into the center of the micelles during the polymerization and self-assembly steps (Kim et al., 2009) (Figure 5).

Recently, González-Miró et al. used an inducible expression of a fusion protein consisting of a *Pha*C and a modified, non-toxic pneumolysin analogue of a *Streptococcus pneumoniae* virulence factor to make PHB beads. This approach elicited a more immunogenic response, as well as an increase in a broad immune response to pneumolysin from various serotypes (González-Miró et al., 2018). Kim et al. used polyhydroxyoctanoate/polyethylene glycol (PHO/PEG) copolymer nanoparticles to nanoprecipitate paclitaxel (an anticancer medication) and found that colon carcinoma in mice was reduced (Kim et al., 2014). The PEGylation of PHA nanoparticles was shown to be crucial to enhance the bioavailability of these drug delivery system in certain applications. PEGylation of a PHA copolymer polyhydroxybutyrate-*co*- hydroxyhexanoate (PHB-*co*-HH) as PEG-PHB-*co*-HH enhanced the delivery of the kinase inhibitor rapamycin (Lu et al., 2014). The aforementioned strategy is particularly intriguing since kinase expression modulation is one of the primary objectives in the prevention of malignant tumor cell growth. Folic acid was coupled to the system in a modification of pegylated PHA-based drug delivery system. The folate receptor has been identified as a therapeutic target to combat tumor growth due to its widespread presence on the surface of cancer cells (Zhang et al., 2010).

According to one report, the first 100 N-terminal amino acid residues of PHA synthase may be deleted without altering the enzyme’s activity (Rehm et al., 2002). As a result, further ligand fusions may be performed without compromising the protein’s natural catalytic activity. In this investigation, various *R. eutropha* mutants were used. PHA synthases using both random and site-specific mutagenesis were synthesized. Four permissive mutants (double and triple mutations) with decreased activity and mutation sites mapping at varied surface-exposed locations were produced from single gene shuffling. Six site-specific mutations were generated to identify amino acid residues that may be important in substrate selectivity. PHA synthase activity was inhibited by replacing residues T323 (I/S) and C438 (G), which are situated in the core structure of the PHA synthase model. Replacement of the two amino acid residues Y445 (F) and L446 (K), which are situated at the surface of the protein model and next to W425, resulted in a decreased activity without affecting substrate specificity, showing that these residues have a functional role. With a surface-exposed location of the mutation, the E267K mutant showed just a modest reduction in activity. Four site-specific deletions were generated in order to investigate the impact of the C-terminus and variant amino acid sequence sequences that connect highly conserved areas. The deleted areas were D281–D290, A372–C382, E578–A589, and V585–A589, but the resulting PHA synthases exhibited no detectable activity, showing that the variable C-terminus and the connecting regions between conserved blocks 2 and 3 as well as 3 and 4 have an important role. Furthermore, the N-terminal region of *P. aeruginosa’s* class II PHA synthase (PhaC_Pa_) and the C-terminal region of *R. eutropha*’s class I PHA synthase (PhaC_Re_) were fused, resulting in three fusion proteins with no observable *in vivo* activity. The fusion protein F1 (PhaCPa-1-265-PhaCRe-289-589), on the other hand, exhibited 13% of wild type *in vitro* activity, with the fusion site positioned at a surface-exposed loop region (Rehm et al., 2002).

In the same context, it was shown that PHB nanoparticles may attach to a solid surface (Paik et al., 2005). These engineered polyesters can be used as suitable tools for anticancer drug delivery and medical imaging procedures. PHA synthase was coupled to a His-tag (10x-histidine) produced in a recombinant *E. coli* strain producing PHB to create a protein–polymer hybrid with His-tag end-functionality. The His-tag was firmly attached to a solid surface derivatized using Ni^+2^-nitrilotriacetic acid (Ni-NTA) (silicone or agarose). All these modifications are good examples of how to synthesize a wide range of protein functionalized PHAs with new characteristics (Paik et al., 2005).

The erosion of PHA surface often starts with the formation of micro-holes at the surface. This allows enzymes and water molecules to attach to the film surface and to initiate the hydrolytic process (Wang et al., 2004). Water molecules first penetrate the amorphous portions of the film, triggering the enzyme-catalyzed hydrolysis of the ester bond (Gumel et al., 2012a). The roughness of the PHA film increases over time throughout the hydrolysis process. While the enzyme is expected to target the amorphous area primarily, wide angle X-ray diffractometry demonstrated a decrease in the crystalline peak after 22 h of processing, indicating that the crystalline region was also hydrolyzed by lipase after the degradation of the amorphous region (Gan et al., 1997). Special surface erosion with PHB depolymerase facilitates the release of 3HB monomers from the surface of P (3HB-*co*-4HB) while leaving nondegradable 4HB monomers on the polymer surface. The degrading characteristics of different polymer constitutions on the polymer surface may differ. The crystallinity, porosity, molecular weight, roughness and monomer composition of the polymer surface are among the factors that contribute to the enzymatic degradation of PHA films (Ansari and Amirul, 2013).

## Functionalization of Polyhydroxyalkanoates

Previously, lipase’s applications were limited since the enzyme was considered to operate best in presence of a high-water content and would rapidly lose activity in organic solvents. Later, it was found that the enzyme is also catalytically active in organic solvents, which aroused the interest of academics and companies because most industrial substrates are naturally hydrophobic (Klibanov, 2001). The use of an organic solvent as reaction medium improves the hydrophobic substrate solubility while also increasing the efficiency of the reaction. Furthermore, a micro-aqueous environment allows reactions that are unachievable in water, such as esterification (Gumel et al., 2011). Organic solvents have been proposed to have a role in extending enzyme activity by substituting water molecules on the enzyme with solvent molecules. Water-based reactions frequently causes downstream challenges during the separation of soluble enzyme and products. However, the enzyme is insoluble in organic solvent environments, which simplifies product recovery. Polymer functionalization may require chemo-enzymatic synthesis processes (Klibanov, 2001). This was demonstrated in the esterification of PCL with hydrophilic moieties in presence of lipase B of *Candida antarctica* (CALB) as a biocatalyst in reaction fluids containing tetrahydrofuran, dichloromethane or dioxane. Longer PHA chains obstruct the enzyme’s ability to interact efficiently with the polymer substrate at the carboxyl end terminal for transesterification. To circumvent this restriction, PCL was hydrolyzed using enzyme prior to chemical transesterification (Cordova, 2001).

The enzymatic step does not only accelerate the next step, but it also assists in the generation of additional carboxyl terminal ends for transesterifications. Gumel et al. (Gumel et al., 2013b) investigated the use of a mixture of two organic solvents, DMSO and chloroform, in a 1:4 ratio to dissolve reaction substrates like PHA and sucrose which exhibit different solubilities. Because a little amount of DMSO was adequate to dissolve enough sucrose and then combine with PHA in chloroform, a system that behaved essentially like a single-phase system could be created (Gumel et al., 2013b). As a result, the consequences of interfacial resistance were avoided. The researchers demonstrated effective functionalization of mcl-PHA with sucrose in this environment by increasing the biodegradability of the modified polymer film. Moreover, Ravenelle and Marchessault described the transesterification of PHB with monomethoxypolyethylene glycol (mPEG) at 190°C in presence of a bis (2-ethylhexanoate) tin catalyst to produce diblock copolymers. However, owing to thermal deterioration that happens during the high temperature process, they were unable to control the molecular weight of the PHB (Ravenelle and Marchessault, 2002).

A novel ceramic-polymer composite with a matrix of tricalcium phosphates and blends of chemically bonded diclofenac and the biocompatible polymer poly (3-hydroxyoctanoate) P (3HO) was recently described. Haraźna et al. found that the hydrophobicity and surface free energy of blends reduced as the proportion of diclofenac modified oligomers increased (Haraźna et al., 2020). The created composites were then employed as a substrate for the pre-osteoblast cell line’s development (MC3T3-E1). *In vitro* biocompatibility testing revealed that the composite containing the lowest concentration of the proposed medication falls within the non-toxic range (viability above 70%). As a result, it might be a perfect new functional bone tissue substitute, enabling not only for the regeneration and healing of the deficiency but also for the inhibition of chronic inflammation (Haraźna et al., 2020).

Gumel et al. (Gumel et al., 2013b) used Novozyme 435 to transesterify PHA to sucrose in an organic solvent combination at a moderate temperature of 50°C. The PDI from the GPC study had a low value of 0.7 with a larger number average molecular weight (Mn) when compared to a low weight average molecular weight (Mw). This suggested that an enzyme-mediated transesterification method might cause a reduced variance in average molecular weight (Gumel et al., 2013b). In another investigation, Novozyme 435 was employed at microaqueous conditions to create a new PHB block copolymer based on PHB-PCL (Dai and Li, 2008). Because of their outstanding thermoplastic characteristics, such block copolymers are considered to have broad biomedical applications (Dai and Li, 2008). The molecular water layer on the enzyme’s surface is the most important component influencing enzyme activity. Unregulated pH, adverse substrate dissolution and reduced conformational stability are the primary reasons of poor activity (Klibanov, 2001). Because the quantity of water held naturally by enzymes becomes the most important element influencing enzyme stability, the solvent hydrophobicity (log P) value is a useful predictor of solvent appropriateness as a reaction medium for the enzyme’s activity.

While the accumulation of the water by-product that reverses the catalysis direction in lipase-catalyzed esterification processes is of significant concern, it has little relevance to other systems that use enzymes such as PHA synthase. For instance, two novel functionalized PHAs containing cyclopropane and chlorine, 3-hydroxy-3-cyclopropylpropionate (3CyP3HP) and 3-hydroxy-4-chlorobutyrate (4Cl3HB), were enzymatically synthesized in an aqueous solution using the PHA synthase from *Escherichia shaposhnikovii* (EsPHAS) (Kamachi et al., 2001).

A recombinant strain of *E. coli* generated the polymerase which was utilized in the *in vitro* polymerization procedures. *R. eutropha* polymerase was also tested for *in vitro* polymerization of the two monomers, however, it was not active with the CoA thioester of 3CyP3HP. With 3CyP3HPCoA and 4Cl3HBCoA, the propagation rates of the two monomers with the EsPHAS polymerase were 1.2 and 6.7 mol of monomer reacted per mole of enzyme catalyst per second, respectively. Furthermore, the PHAs obtained had number-average molecular weights of 371,000 and 189,000, respectively (Kamachi et al., 2001).

PHAs are soluble in chlorinated and other organic solvents at room temperature, including chloroform, chloropropane, carbon tetrachloride, dichloromethane, dichloroethane, 1,2-propylenecarbonate, hot acetone, tetrahydrofuran and toluene. The advantages of using aqueous organic solvents instead of non-aqueous systems for lipase-catalyzed PHA modifications include increased solubility of nonpolar substrates, the enzyme favoring ester-bond synthesis rather than hydrolysis, and the elimination of microbial growth that typically gives contaminations of the aqueous reaction mixture (Kunasundari and Sudesh, 2011). Several solvent systems have been investigated including a hydrophobic organic solvent (monophasic), a water-and-hydrophilic solvent (monophasic), a water-and-water immiscible (two phase) solvent and a nearly dry organic solvent system. A monophasic system has the benefit of having lowest diffusion resistance between the substrate in the hydrophilic solvent and the water phase, as well as maximal dissolution of substrate concentration, which results in an increase of the reaction rate (Ogino and Ishikawa, 2001).

The polymer backbone chains aggregate together in crystalline PHA, leaving the alkyl side chains facing outward and covering the polymer surface. Once the polymers are dissolved in a solvent, the solvent molecules diffuse through the polymer matrix until they reach the polymer core and stretch the polymer backbones, resulting in a swelled and solvated mass. Then it disintegrates, and the polymer chains begin to disperse into a real solution. The polymer backbones and both functional groups are now susceptible to an attack of the enzyme (Stevens, 2009). Direct contact between an enzyme and organic solvent causes preferred partition of the enzyme’s hydrophobic component towards the solvent, as well as alterations in enzyme structure, which can finally lead to enzyme deactivation. When a two-phase system is used to reduce enzyme molecule inactivation, denaturing in the interphase between water and organic phase may still occur, but at a reduced frequency (Ogino and Ishikawa, 2001).

## Degradation Products

Polymer degradations are defined as physical, chemical and biological modifications or combinations that occur during processing and storage, leading to the breakage of polymeric backbone bonds. Shorter oligomers, monomers and other breakdown products are therefore be produced.

### Polyhydroxyalkanoate as Source for Chiral Monomer

Because of their well-known biological properties, there has been an increase in demand for pure biodegradable PHA enantiomers. The widespread use of enantiomeric pure compounds in areas like agriculture, food and medicine, increases the demand for large-scale manufacturing (Chen and Wu, 2005). (*R*)-(–)- Hydroxycarboxylic acid is a chiral building block that may be used for synthesis of fine compounds like aromatics, vitamins, pheromones and antibiotics. Its carboxylic and hydroxyl groups can be modified and used as a precursor for the production of novel molecules. PHA synthase from *R. eutropha* was utilized in a different study to polymerize PHB and PHV on hydrophobic highly oriented pyrolytic graphite (HOPG) and alkanethiol self-assembled monolayer (SAM) surfaces, which are used to support other functional biomolecules including biotin and streptavidin. Surface modifications have been linked to biological and biotechnological applications (Sato et al., 2008). This enzymatic polymerization of PHA on two hydrophobic surfaces, HOPG and SAM was used to prepare *R*-enantiomer monomers [(*R*)-3-hydroxyacyl-CoA] with an acyl chain length of four to six carbon atoms. PHA homopolymers with different side-chain lengths, poly [(*R*)-3-hydroxybutyrate] [P (3HB)] and poly [(*R*)-3-hydroxyvalerate] [P (3HV)] were successfully synthesized from such *R*-enantiomer monomers on HOPG substrates. Thus, the surface morphologies were analyzed and revealed a nanometer thick PHA film. These properties will enhance the polymeric film formation through decreasing the film thickness and increase the film elasticity (Sato et al., 2008).

The enzymatic polymerization of PHA on two hydrophobic surfaces, a HOPG and an alkanethiol SAM, was investigated *in vivo* and *in vitro* using atomic force microscopy (AFM) and quartz crystal microbalance (QCM), using the *R. eutropha* PHA synthase (PhaC_Re_) purified from soluble protein fractions HisTrap HP columns a biocatalyst. *R*-enantiomer monomers [(*R*)-3-hydroxyacyl-CoA] with an acyl chain length of four to six carbon atoms were prepared using a (*R*)-specific enoyl-CoA hydratase. On HOPG substrates, PHA homopolymers with differing side-chain lengths, P (3HB) and P (3HV), were effectively synthesized from such *R*-enantiomer monomers. AFM was used to examine the surface morphologies after the reaction, revealing a nanoscale thick PHA layer. On an alkanethiol (C18) SAM surface produced on a gold electrode employing QCM, the same biochemical polymerization process was observed. On the hydrophobic surface, a complicated sequence of PhaC_Re_ adsorption and PHA polymerization occurred. The potential mechanisms of the PhaC_Re_-catalyzed polymerization process on the surface of hydrophobic substrates were postulated based on these results (Sato et al., 2008).

Using high-speed scanning atomic force microscopy (HS-AFM), the early step of *in vitro* PHA polymerization by PHA synthase from PhaC_Re_ on a mica substrate in water was detected. The adsorption–desorption cycle of the PhaC_Re_ molecule on mica was monitored in real time before PHA polymerization. There was no substantial change on the mica substrate for about 30 s following the addition of the PHA monomer, although PhaC_Re_ might be converted into an active enzyme in water upon contact with the monomer during this time (Ushimaru et al., 2017). Following that, linearly elongating rod-shaped structures appeared on the mica substrate, which might have been caused by the polymerization reaction. The height of these elongating items was far more than that of a single PHA chain. This finding shows that the PHA chains formed in the presented studies may have a semiregular structure (Ushimaru et al., 2017).

### Polyhydroxyalkanoate as Source for Oligomers

PHA oligomers have attracted a lot of attention due to their *in vivo* bioresorbability and biodegradability. Likewise, dimers and trimers of 3HB may be quickly converted to monomers in rat and human tissues. As a result, several types of dendrimers based on oligo-HA may have been produced and employed for a variety of drug delivery applications (Chen and Wu, 2005). Oligomers derived from the degradation of purified PHA can be utilized to graft copolymers at the carboxylic end terminal. When compared to pure PHA, oligomers have a narrower molecular weight dispersion and are more functional. Grafting oligomers enables a better regulated procedures as well as a high yield (83%) (Nguyen and Marchessault, 2004).

The first report on the production and copolymerization of PHB methacrylic macromonomers holds up the possibility of novel biomaterial characteristics. The single glass transitions observed for each copolymer composition showed that the residues were randomly arranged and that the macrodomains were in nano scale. PHB grafts should be split into tiny crystallites and mixed with poly (methyl methacrylate) (PMMA) segments to produce noncrystalline areas; these areas are responsible for glass transitions. With an increasing PHB macromonomer concentration, the crystallinity increased gradually. As a result, the microtexture of the copolymers is rather homogeneous, and strain resistance is dependent on both noncrystalline and crystalline, domains. The latter can serve as links between chains, providing continuity and pseudo-crosslinking to the overall structure. These comb-like co-polymers are predicted to lose weight slowly owing to biodegradation and hydrolytic erosion of the PHB side chains; nevertheless, the vinyl backbone should remain intact in both situations. PHB graft copolymers should also exhibit a better biocompatibility. In general, the diverse family of potential macromonomers (PHAs) should provide a variety of mechanical and surface properties for implant and bone cement applications (Nguyen and Marchessault, 2004).

Recently, the secretion of the intracellularly produced (*R*)-3-hydroxybutyrate oligomer (3HBO) from recombinant *E. coli* cells was established using a new growth method. The authors tried to develop microbiological 3HBO with a diethylene glycol terminus (3HBO-DEG) as a macromonomer for polymeric materials. They developed a recombinant *E. coli* strains with genes encoding PHA synthases (PhaC, PhaEC, or PhaRC) that can insert chain transfer (CT) agents like DEG into the polymer’s terminal and make CT end-capped oligomers. To do this, each strain was grown in DEG-supplemented conditions, and 3HBO-DEG synthesis was validated. As a result, the PHA synthase developed from *B. cereus* YB-4 (PhaRC_YB4_) had the largest secretory synthesis of 3HBO-DEG (Hiroe et al., 2021).

## Chemical Modification of Polyhydroxyalkanoates

**FIGURE 6:**
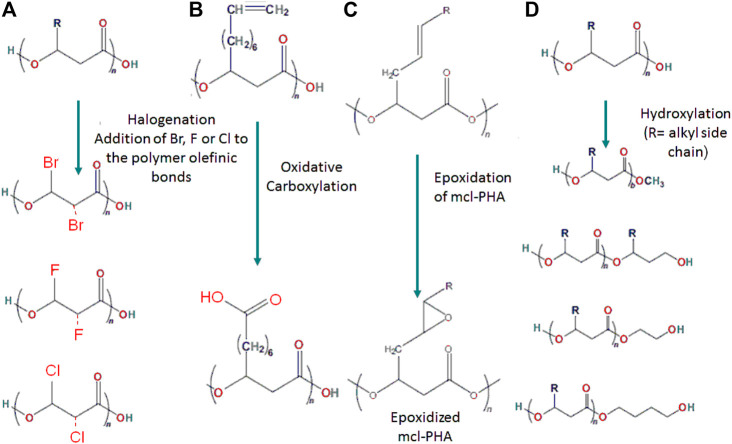
Structural illustration showing the chemical polymeric modification through **(A)** Halogenation, **(B)** Carboxylation, **(C)** Epoxidation and **(D)** Hydroxylation.

Structures of PHAs can be chemically changed to generate a modified polymer with predictable variations in functionality and molecular weight. Chemical reactions enable the mass manufacturing of a homogeneous product as well as the integration of various functional groups to generate usable tailor-made polymers with desirable characteristics for specialized applications. PHAs can be modified chemically using a variety of techniques such as halogenation, carboxylation, epoxidation, and hydroxylation (Figure 6).

### Halogenation

PHA halogenation is regarded as an important technique to broaden polymer functionalities and applications. Through an addition process, halogen atoms such as bromine, fluorine, and chlorine were introduced to the olefinic bonds of unsaturated PHA. In addition, these halogens were added to saturated PHA by substitution reactions (Arkin and Hazer, 2002). For example, to create chlorine gas, extra HCl was added dropwise to KMnO_4_. The gas was then passed through a solution of sticky unsaturated PHA obtained from *P. oleovorans*. At a chlorine concentration contributing to about 54% of the weight of the polymer, this resulted in a rigid and crystalline polymer (Arkin et al., 2000). The chlorinated PHA (PHA-Cl) has higher glass transition and melting temperatures (*T*_*g*_ = 58°C, *T*_*m*_ = 125°C) than pure PHA (*T*_*g*_ = 50°C, *T*_*m*_ = 55°C) depending on the chlorine concentration. However, hydrolysis of the polymer backbone was detected as the chlorine concentration of the PHA-Cl increased, owing to the decreased molecular weight of the PHA-Cl (Arkin et al., 2000).

In another research, quaternary ammonium salts, thiosulfate moieties, and phenyl derivatives from PHA-Cl were synthesized. The Friedel–Crafts process was used to cross-link the modified PHA-Cl with benzene through electrophilic aromatic substitution. By comparing the respective peak areas of the methine protons at the polymer backbone, the random composition of PHA-Cl was determined from the ^1^H NMR spectra (Arkin and Hazer, 2002). As a result of enhanced chlorination of the methyl protons, the peaks of methyl protons were shifted from 14 and 15.8 ppm to 20–25.5 ppm. Furthermore, the mole fractions of PHA-Cl were determined by comparing the peak regions of protons on chlorinated α-carbons with protons on β-carbons (Arkin and Hazer, 2002).

New radiopaque biodegradable and biocompatible iodinated polymers based on PHB have recently been discovered. The hydroxyl ends were capped with 4-iodobenzoic acid and 2,3,5-tri-iodobenzoic acid after diethanol amine was attached to PHB. Tri-novel radiopaque polymers were created in this way. They were tested to see whether they might be used as radiopaque implant biomaterials that could indicate X-ray visibility in a noninvasive way utilizing standard X-ray absorption imaging procedures (Erol et al., 2020). *In vivo*, these polymers showed excellent radiopacity using standard X-ray imaging methods. Furthermore, the biocompatibility of these iodinated polymers was assessed. On the surgical site, there were no evidence of infection or abscess development. These new radiopaque PHBs have the potential to be excellent biomaterials for next-generation radiopaque materials (Erol et al., 2020).

In the same context, *P. putida* generated PHA with fluorinated phenoxy side groups when fluorophenoxyalkanoic acids were employed as carbon sources. NMR and GCMS studies verified the synthesis of homopolymer poly (3-hydroxy-5-(2,4-difluorophenoxy) pentanoate). Fluorine atoms located in the PHA’s side chain had a significant impact on its physical characteristics. In contrast to other mcl-PHAs, this fluorinated PHA was cream-colored, opaque and had a higher melting point (100°C) as well as increased crystallinity. The study of surface contact angles indicated that the PHA with two fluorine atoms has water repellent characteristics. Cell development was impacted by the amount of substituted fluorine atoms in the carbon source and difluorine-substituted phenoxyalkanoic acids decreased cell growth and polymer synthesis when compared to non-substituted phenoxyalkanoic acids (Takagi et al., 2004).

### Carboxylation

Carboxylation of PHA is the addition of a carboxylic group to the polymeric monomer. Carboxylic groups in polymers often serve as functional binding sites for bioactive moieties such as probes for targeting proteins and hydrophilic components (Kai and Loh, 2014). The addition of these functional groups to PHAs adds important properties that bioconversion mechanisms cannot readily attain. These chemically modified PHAs with enhanced characteristics can be used as multifunctional materials. According to research, chemical modification was used to regulate the hydrophilic/hydrophobic balance of PHA materials, and the best products for hydrolysis are PHAs with carboxylic groups in the side chains, such as poly (3-hydroxyoctanoate-*co*-9-carboxy-3-hydroxydecanoate) and its derivatives (Renard et al., 2007). Furthermore, by modifying unsaturated bacterial polyesters chemically, copolymers of PHO containing carboxylic groups in side chains have been produced. The oxidation of the pendant alkenes is complete, and a significant decrease in polymer molecular weight was observed. The inclusion of repeating units with pendant carboxy groups in a 25% proportion has increased the hydrophilicity of these novel polymeric structures (Kurth et al., 2002). Thus, the carboxylation of PHA improves the polymer’s hydrophilicity by increasing water penetration, which is facilitated by ester group hydrolysis by water (Kurth et al., 2002; Renard et al., 2007).

To carboxylate PHA with an unsaturated group, a chemical oxidative method is often used (Renard et al., 2007; Kai and Loh, 2014). Kurth et al. and Lee and Park improved this technique by using crown ether as a phase transfer and dissociating agent for the KMnO_4_ to carboxylate the unsaturated poly (3-hydroxy-10-undecenoic-*co*-3-hydroxyoctanoate) (PHOU) (Lee and Park, 2000; Kurth et al., 2002). PHAs with pendant carboxyl groups were created by chemically modifying unsaturated PHAs with KMnO_4_ at 55°C, despite a significant loss in PHA molecular weight during the process. The reduction of molecular weight was related to either macromolecular chain breakdown or variations in hydrodynamic radii between the carboxylated polymer and size-exclusion chromatography (SEC) polystyrene standards during the procedure (Kurth et al., 2002). The presence of carboxylic groups in the products was confirmed by IR and ^1^H NMR spectra. The degree of carboxylation increased to about 50% after 2 h of reaction time, but it did not increase further upon longer incubation. The polymers with a carboxylation degree of 40–50% are fully soluble in water/Na_2_CO_3_, suggesting that the modified PHAs have significantly increased hydrophilicity (Lee and Park, 2000).

The disappearance of the ^1^H NMR signals (4.9 and 5.7 ppm) ascribed to the double bond allowed the scientists to quantify the oxidation. Although the double bonds were completely oxidized, the researchers discovered that 25% carboxylation of PHOU was sufficient to increase the polymer’s hydrophilicity (Kessler et al., 2014). The first step was to utilize UV-assisted surface modification in presence of oxygen to accomplish controlled breakdown of PHB films by entomopathogenic fungi. Surface analysis techniques were used to investigated the treated surfaces (FTIR in Attenuated Total Reflectance mode, Near-edge X-ray Absorption Fine Structure, SEM, Optical Microscopy, X-ray Photoelectron Spectroscopy, Gel Permeation Chromatography, water contact angle and weight loss). XPS and NEXAFS spectroscopy showed the presence of novel carbonyl groups in new chemical contexts following UV-assisted treatments. The oxidizing environment prevented the production of C=C bonds, indicating that the Norrish Type II mechanism is inhibited during or as a result of the treatments (Kessler et al., 2014). The increased hydrophilicity and concentration of oxygenated functional groups at the surface of the treated films may have enhanced biodegradability. This straightforward approach may be utilized to enhance and regulate the deterioration rate of PHB films in applications requiring a controllable degradation rate.

Stigers and Tew created a novel synthetic procedure that employs osmium tetraoxide and potassium peroxymonosulfate (oxone) in dimethylformamide to solve the issue of molecular weight loss during carboxylation. The oxidation procedure was carried out at 60°C for 8 h with negligible polymer degradation at the end. NMR, IR and GPC were used to validate this little backbone breakdown. The polymer’s solubility before and after reaction was significantly varied and the investigations were done using several solvents including acetone, tetrahydrofuran (THF) and water. Changes in the hydrophobicity of the resulting product are substantial as observed by solubility in H_2_O and organics solvent mixtures. The modified PHA’s carboxylic group was found to be useful in the production of block and grafted copolymers (Stigers and Tew, 2003). Additionally, to graft modified PHA and linoleic acid onto chitosan, condensation processes between carboxylic acids and amine groups were used. Unreacted PHAs and linoleic acid were removed using chloroform extraction, while unreacted chitosan was removed using a 2 wt% acetic acid solution. ^13^C-NMR (in solid state), FTIR, TGA and DSC were used to characterize the pure chitosan graft copolymers. The proportion of microbial polyester grafted onto chitosan backbone varied from 7 to 52 wt% as a function of PHA molecular weight or function of steric effect (Arslan et al., 2007). Graft copolymers were soluble, partly soluble or insoluble in 2 wt% acetic acid, depending on the number of free primary amine groups on the chitosan backbone and the degree of grafting. Thermal studies of PHO-*g*-Chitosan graft copolymers revealed that the melting transitions *T*_*m*_S at 80, 100, and 113°C or a wide *T*_*m*_S between 60.5–124.5°C and 75–125°C, whereas pure chitosan revealed a sharp *T*_*m*_ at 123°C (Arslan et al., 2007). Moreover, Babinot et al. utilized click ligation to esterify the pendant–COOH of carboxylated PHA with propargyl alcohol, resulting in a clickable-alkyne group that was then copolymerized onto the modified PHA using poly (ethylene glycol) (PEG) macromer (Babinot et al., 2011). The authors reported the modular synthesis of scl and mcl poly (3-hydroxyalkanoate)s-*b*-poly (ethylene glycol) (PHAs-*b*-PEG) diblock copolymers. First, heat treatment yielded length-controlled oligomers of hydrophobic Poly (hydroxybutyrate-*co*-Hydroxyvalerate) (PHBHV), Poly (3-hydroxybutyrate-*co*-3-hydroxyhexanoate) (PHBHHx), and poly (3-hydroxyoctanoate-*co*-3-hydroxyhexanoate) (PHOHHx) containing a carboxylic acid end group, with molar weights ranging from 3,800 to 15,000 g mol^−1^. Following quantitative functionalization with propargylamine, a 5,000 g mol^−1^ ligation with azide-terminated PEG was achieved utilizing the copper (I) catalyzed azide alkyne cycloaddition (CuAAC). With molar weights ranging from 9,900 to 23,100 g mol^−1^, well-defined diblock copolymers were produced with up to 93% yield (Babinot et al., 2011).

Using the PHA biosynthetic operon from *B. cereus* 6E/2, several *E. coli*-based production methods were constructed to increase the accumulation of PHAs with high mcl moieties. The use of media optimization and system engineering resulted in the generation of up to 260 mg/L of PHAs. The analysis of the polymers indicated a low level of crystallinity and outstanding hydrophobic properties. A unique enzyme-based method was devised for further functionalization (Vastano et al., 2017). CaLB catalyzed the terminal coupling of PHA with: I dimethyl itaconate (DMI) to introduce reactive side chain vinyl moieties for simple coupling of functional molecules, and/or 2) biocompatible polyethylene glycol (PEG) to regulate polymer hydrophilicity. This study’s functionalized DMI-PHA, PEG-PHA, and PEG-DMI-PHA polymers, which were produced and characterized by NMR, GPC, and FT-IR, thereby offering new possibilities for the use of PHAs as biodegradable and biocompatible materials of choice for biomedical applications (Vastano et al., 2017).

### Epoxidation

The need for a strong reactivity of epoxide groups under moderate conditions has been identified as a key element for mcl-PHA modifications. Without causing polymer degradation, the epoxide group can engage in a variety of processes such as cross-linking to attach a bioactive moieties, an ionizable group or copolymers (Imamura et al., 2001). Several studies have demonstrated effective epoxidation-based chemical modification of PHA (Park et al., 1998a; Park et al., 1998b). Park et al. used m-chloroperoxybenzoic acid (m-CPBA) to epoxidize PHOU with a regulated number of olefinic linkages. The process followed second-order reaction kinetics regardless of the amount of polymeric olefinic groups, with an observed starting reaction rate of 1.1 × 10^−3^ L mol^-1^ s^-1^ at 20°C. *T*_*m*_ and melting enthalpy, on the other hand, were shown to decrease as the olefinic linkages were converted to epoxy groups. Interestingly, regardless of the PHOU composition employed, the scientists found a rise in *T*_*g*_ of around 0.25°C for each 1 mol% of epoxide group (Park et al., 1998a). Similarly, the researchers cross-linked epoxidized PHOU with succinic anhydride. The process was initiated by 2-ethyl-4-methylimidazole and carried out at 90°C for 0.5–4 h, yielding a highly elastic crosslinked PHA. They discovered that by performing the process under moderate acidic conditions, they were able to prevent the observed polymer degradation (Park et al., 1998b).

Park et al. discovered that the thermal stability of the epoxidized PHA improves with increasing epoxy group regarding the thermal stability. The observed improvement in thermal stability was attributed to intermolecular thermal cross-linking interactions between the pendant epoxy groups and the carboxylic acid groups produced by β-elimination of the polymer random chain. This conclusion was obtained from the emergence of a thermal exothermic peak at 375°C in the DSC thermogram of the epoxidized polymer, followed by an endothermic melting temperature peak at 299°C (Park et al., 1999). In addition, mcl-PHA derived from linseed oil was found to have a large amount of olefinic side chains, making the polymer viscous and sticky at room temperature (Ashby et al., 2000). Except as a bio-adhesive, the polymer has limited potential uses; however, the range of applications can be increased by increasing the rigidity and stiffness of the polymer. In order to improve the mcl-PHA cross-linking capacity, Ashby et al. utilized m-CPBA to convert about 37% of olefinic bonds in linseed oil generated mcl-PHA side-chains to epoxy groups (Ashby et al., 2000). The presence of an epoxide chemical shift at 58 ppm in the ^13^C NMR spectra of both the pure mcl-PHA and the epoxidized mcl-PHA verified the polymer epoxidation. The poor olefinic conversion yield was due to steric hindrance induced by the closeness of the internal olefins to the side chains. It was proposed that the intermolecular crosslinking occurred via ether cross-links. It was hypothesized that in this form of cross-linking, a nucleophile or radical species first opened the carboxy-oxirane ring, causing electron rearrangement and the production of an alkoxide anion, which then started a nucleophilic assault on another epoxidized PHA oxirane carbon (Ashby et al., 2000). When exposed to air, epoxidation boosted the cross-linking of the mcl-PHA, leading in a rise in tensile strength and Young’s modulus from 4.8 to 20.7 and 12.9–510.6 MPa, respectively. As a result, the modified PHA has a shorter (25-days) stiffness in comparison to the comparable neat PHA, which has a longer (50–75-days) stiffness (Ashby et al., 2000).

A combination of PHA and malic anhydride (PHA/MeA) crosslinked with tea plant fiber (PHA-*g*-MeA/t-TPF) was tested in another investigation. Due to its excellent compatibility with TPF, PHA-*g*-MA/t-TPF has improved mechanical characteristics in comparison to PHA/TPF (Wu, 2013). Due to ester linkage formation, t-TPF showed more dispersion homogeneity in the matrix of PHA-*g*-MeA, resulting in the production of cross-linked and branching bigger molecules between the–COOH and OH groups of PHA/MeA and t-TPF, respectively. With an increase in TPF content, this crosslinked composite had more polished qualities such as higher water resistance, easier processing due to low viscosity, and high biodegradability (Wu, 2013).

Another study was also conducted to fabricate an unsaturated composite PHB/PHBU generated by an *E. coli* strain, which was then cross-linked using thiolene click chemistry and examined for improved physical attributes and biocompatibility with human mesenchymal stem cells. There was a considerable increase in tensile strength, which was consistent with a material exhibiting the qualities needed for soft tissue replacement (Levine et al., 2015). In the same study 52, it was also discovered that after cross-linking, this chemically modified substance was not hazardous to human cells. As a result, crosslinking and epoxidation are related, and when combined, they provide an effective method for modifying PHAs (Levine et al., 2015).

In a different study Lee et al. reported on the successful cross-linking of epoxidized PHO using hexamethylene diamine (HMDA) as a cross-linker in a process carried out at 90°C for 0.5–24 h. They found that the degree of crosslinking has a substantial impact on the modified copolymer’s *T*_*g*_ and the relative storage modulus (Lee M. Y. et al., 1999). Furthermore, the effect of cross-linking on the thermal stability of epoxidized PHA was investigated by testing the cross-linking between the PHA and HMDA (in the absence of a catalyst) or between PHA and succinic acid (in the presence of a catalyst). According to the findings, increasing the quantity of basic catalyst or of the diamine cross-linker resulted in a decrease in the thermal stability of cross-linked PHA. The finding was attributed to basic ester cleavage of the polymer backbone or cross-linker amine groups catalyzed by the basic catalyst (Lee and Park, 1999). In another study, Bear et al. used m-CPBA to chemically epoxidize mcl-PHA derived from *P. oleovorans* and *Rhodospirillum rubrum* (a pink-coloured, Gram-negative Proteobacterium). The ^1^HNMR analysis indicated 36.7% epoxidation when the signals of α, β-oxirane proton (2.75 and 2.9 ppm) were compared to the signals of methylene protons (2.6–2.5 ppm) in the PHA backbone (Bear et al., 1997).

### Hydroxylation

Hydroxylation has been shown to alter the characteristics of PHA and their copolymers. In most cases, acid or base-catalyzed reactions were utilized for the hydroxylation of PHA in presence of low molecular weight mono or diol molecules. The relevance of hydroxy-terminated PHA in block copolymerization cannot be overstated. PHA methyl esters with monohydroxy-terminated groups were produced by methanolysis of PHA (Zhou et al., 2012; Kwiecień et al., 2013). Timbart et al. used both base and acid-catalyzed hydrolyses to produce monohydroxylated oligomers of PHO and PHOU. An alcoholic solution of NaOH was used to accelerate basic hydrolysis at pH 10–14, and the process was terminated by adding strong aqueous hydrochloric acid (Timbart et al., 2007). The acid hydrolysis, on the other hand, was carried out in two ways: 1) monohydroxylation by acidic (sulfuric acid) methanolysis at 100°C, which yielded the respective 3-hydroxymethyl esters bearing a methyl protected carboxylic acid, and 2) a reaction catalyzed by para-toluenesulfonic acid monohydrate (PTSA) at 120°C, which was stopped by cooling the mixture in an ice bath (Timbart et al., 2007). PHO ester linkages were shown to be stable at pH 10–12. Increasing the pH to 14 resulted in a faster hydrolysis rate and the formation of oligomers with unsaturated end groups as a consequence of a McLafferty rearrangement. The study determined that an acid-catalyzed reaction and methanolysis produced more PHO oligomers than basic hydrolysis. Moreover, the kind of solvent employed such as toluene or dichloroethane had an effect on the reported decrease in polymer molecular weight (Timbart et al., 2007).

A series of innovative amphiphilic block polyurethanes (PUHE) has been prepared successfully using a solution polymerization of the derived PHB-diol and poly (ethylene glycol) with a coupling agent of 1,6-hexamethylene diisocyanate (HDI), while the PHB-diol was prepared via PHB and ethylene glycol transesterification. Nonaqueous titration revealed that the hydroxyl content of PHB-diols ranges from 1.36 to 1.99 (molar ratio). GPC, ^1^H NMR, and FTIR were used to analyze the molecular weight and chemical contents of PUHE and PHB-diol in depth, confirming the effective synthesis of PUHE (Xue et al., 2016). Because PUHE contains 33% PHB, its tensile strength and elongation at break might be as high as 20 MPa and 210%, respectively. TGA curves show that block-bonding between PHB-diol and PEG improves PHB-heat diol’s stability. Weight loss and SEM were used to investigate PUHE film deterioration. It was found that deterioration happened gradually from the outside to inside, and that the pace of degradation could be regulated by altering the PHB/PEG ratios. Because of these characteristics, PUHE can be utilized as a biodegradable thermoplastic elastomer (Xue et al., 2016).

Polymeric chain breakage was shown to occur more frequently in toluene than in dichloroethane, which was attributed to the polymer’s greater solubility in toluene. P-toluenesulfonic acid (PTSA) was used to catalyze the monohydroxylation of poly (3- hydroxybutyrate-*co*-4-hydroxybutyrate) (P3HB4HB) in the presence of methanol (Zhou et al., 2012). The modified PHA was acrylated and grafted onto poly (ethyleneimine) by Michael addition, resulting in a material that was utilized to transport siRNA. A bi-functional telechelic PHA as a macro-initiator is often required in the production of PHA tri-block copolymers. Low molecular weight diols are employed as a micro-initiator of the PHA-diol reaction in this method. During the process, the diol’s hydroxyl groups were hypothesized to randomly break the polymeric ester bonds, resulting in the dihydroxy terminated PHA. In the presence of ethylene glycol (micro-initiator), dibutyltin dilaurate was employed as catalyst to create an enantiomerically pure telechelic dihydroxy-terminated PHO and its copolymers at 80–91% yield (Andrade et al., 2002).

The modified telechelic PHO-diol was discovered to have lower *T*_*g*_ and *T*_*m*_ than pure PHO. However, this observation was in contradiction the previously observed improvement in thermal stability of di-hydroxy terminated P3HB produced by microbial fermentation (Shah et al., 2000). Chen et al. used 1,4-butanediol and PTSA to produce modified P3HB4HB and PHBHHx diols that were grafted with poly (ester urethanes) through Michael addition. Melting polymerization (MP) with 1,6-hexamethylene diisocyanate (HDI) as a coupling agent was used to create it. The synthesized poly (ester urethanes) films were thoroughly characterized using ^1^H-NMR, FT-IR, GPC, DSC, mechanical properties, static water contact angles, cell proliferation using smooth muscle cells from rabbit aorta (RaSMCs) and immortalized human keratinocytes (HaCat) and blood coagulation behavior. DSC analysis revealed that poly (ester urethanes) samples exhibited poor crystallinity at ambient temperature and were completely amorphous following a melt-quenched procedure (Chen et al., 2009a).

Additionally, Kwiecień et al. reported a novel technique for P3HB4HB hydroxylation utilizing lithium borohydride (LiBH_4_) for selective partial breakdown of the polymer ester linkages. After the polymer had been fully solubilized in tetrahydrofuran, the reaction was carried out by drop-wise addition of 2M LiBH_4_ at a temperature of ˂ 20°C. About 97% of pure modified PHA oligodiols were produced after a series of purification procedures involving chloroform and water washing, followed by evaporation of the organic phase (Kwiecień et al., 2013). The applied reduction procedure of the PHA oligodiols was extremely selective, as indicated by NMR and electrospray ionization-mass spectrometry (ESI-MS) studies. The technique, according to the researchers, is flexible and relevant for the synthesis of any PHA oligodiol. A reaction between the polymer hydroxyl group and the–NCO group of aliphatic l-lysine methyl ester diisocyanate (LDI), which was used as a crosslinker, was used to create block copolymers of PHO and poly (ester-urethanes) (Kwiecień et al., 2013). Due to the lack of the toxic effect, mutagenic, and carcinogenic aromatic amine degradation byproducts of aromatic diisocyanate-derived polyurethane, aliphatic diisocyanate (LDI) was chosen over aromatic diisocyanate.

Similarly, Chen et al. utilized simple melt polymerization to create a block poly (ester-urethane) based on diols of P3HB4HB and poly (3-hydroxyhexanoate-*co*-3-hydroxyoctanoate) (P3HHx3HO) using the coupling agent 1,6-hexamethylene diisocyanate (HDI) (Chen et al., 2009b). The platelet adhesion and lactate dehydrogenase (LDH) assays demonstrated that the synthesized material had considerably greater platelet adhesion than the raw polymers, and even higher than polylactic acid (PLA) and PHB. Indeed, this kind of poly (ester-urethane) has been found to have the characteristics necessary for usage in medical applications, such as improved wound healing activity. A significant number of PHA-based products were created from hydroxylated PHA, and they have been thoroughly studied (Chen et al., 2009b).

### Chemical Modification of Polyhydroxyalkanoates’ Side Chain

**FIGURE 7:**
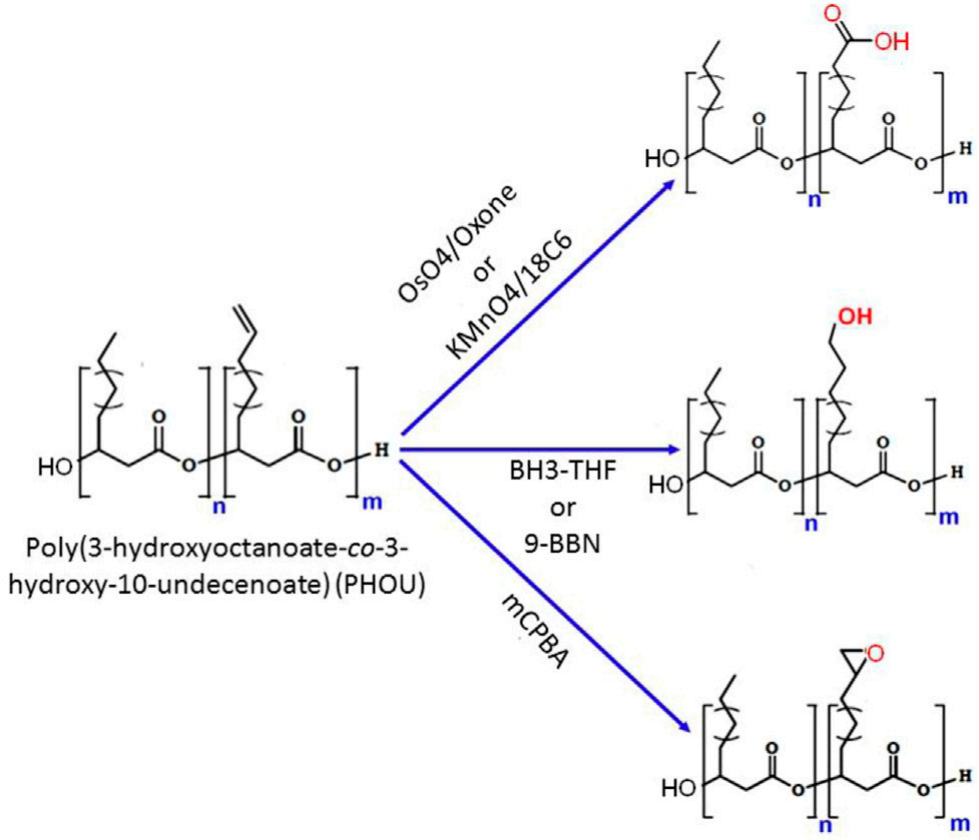
Chemical scheme of different methods for the modifications of poly (3-hydroxy-10-undecenoic-*co*-3-hydroxyoctanoate) (PHOU) side chain.

PHA biosynthesis provides a wide range of sidechain functions, although not all functional groups may be produced by the biotechnological processes. The most typical synthetic route to functionality in natural PHAs is to manufacture a polymer with unsaturated side chains such as PHOU, followed by modifications at the double bond to provide the required functional group (Michalak et al., 2017). The most common way to functionalize the unsaturated side chains of PHAs is to oxidize them to epoxide, carboxyl, or hydroxyl groups. PHA epoxidation is a helpful technique for modifying the unsaturated side chains. One such example is the epoxidation of PHOU containing 20–100% 10-undecenoic acid with m-chloroperbenzoic acid (mCPBA), which results in poly (3-hydroxy-10-epoxyundecanoic-*co*-3-hydroxyoctanoate) (PHOE) (Figure 7) (Bear et al., 1997). When a 2-fold molar excess of oxidizing agent was employed, the transition of the double bond to an oxirane was quantifiable. For PHOU with 5–95% unsaturated side chains and relatively few hexanoate and nonenoate units, increasing the number of unsaturated side groups lowered the reaction time required to quantitatively epoxidize the polymer. The epoxidation of PHOU raised the *T*_*g*_ by 0.25% for every 1 mol% of epoxide concentration (Park et al., 1998a; Park et al., 1998b).

Even with a low epoxide-group concentration of 5%, crosslinking PHOE with succinic acid anhydride produced a gel fraction of >65%. Although the glass transition temperatures rose for PHOE samples with more epoxide groups, the differences were negligible. PHOEs with 10, 22, and 34 mol% epoxide groups were also crosslinked with hexylmethylene diamine. The resulting materials have a higher *T*_*g*_ and relative storage moduli (Lee M. Y. et al., 1999). The distance between the double bond and the PHA backbone had no effect on the epoxidation process. The epoxidation of poly (3-hydroxybutyrate-*co*-3-hydroxyvalerate-*co*-3-hydroxy-pentenoate) [P(HB-HV-HPE)] containing 10% 3-hydroxy-4-pentenoic acid (HPE) repeating units produced comparable results to PHOU (Bear et al., 1997). Similarly, epoxidation of poly (3-hydroxy-4-pentenoate) homopolymer with mCPBA yielded poly (3-hydroxy-4-pentenoate-*co*-3-hydroxy-4-epoxyvalerate) [P (3HPE-*co*-3HPO)] with 45% 3-hydroxy-4-epoxyvalerate units, resulting in an elevated glass transition temperature of the produced copolymer (Valentin et al., 1999).

Cationic PHA such as poly (3-hydroxyoctanoate)-*co*-{3-hydroxy-11-[bis(2-hydroxyethyl)-amino]-10-hydroxyundecanoate} (PHON), was generated by epoxidizing PHOU and then combining the epoxy groups with diethanoloamine (Sparks and Scholz, 2008). This polyester is soluble in solvents such as DMSO, Dimethylformamide (DMF) and even water, but it is insoluble in organic solvents such as methylene chloride, THF or chloroform. The basic chain of the PHA remained stable during the epoxidation process, but the interaction with the amine resulted in breakdowns of the polymer backbone, as indicated by the reduction in product molecular weight. Later, the use of cationic PHON as a plasmid for a DNA delivery method was shown to be promising. Sparks et al. described PHON for application as a plasmid for DNA delivery. PHON was found to attach to DNA and compress it into positively charged particles smaller than 200 nm in size. PHON has been demonstrated to preserve plasmid DNA against nuclease degradation for up to 30 min in this manner. Furthermore, *in vitro* treatment of mammalian cells with PHON/DNA complexes resulted in luciferase production as a result of gene delivery. The ability of PHON to transfect mammalian cells *in vitro* was also examined. The expression of luciferase in African green monkey kidney cells (COS-1) was tested using PHON formulations containing luciferase plasmid (Sparks and Scholz, 2009).

Furthermore, the production of PHA with carboxyl side groups may be performed by oxidizing the double bond in PHOU (Figure 7) with KMnO_4_ in presence of acetic acid and 18-crown-6 ether. Using this method, poly (3-hydroxyoctanoate-*co*-3-hydroxy-9-carboxy-decenoate) (PHOD) with 10 and 25% carboxyl groups was synthesized. The molecular weight of 10% PHOD-COOH remained nearly constant, but the molecular weight of 25% PHOD-COOH decreased significantly owing to a hydrolytic breakdown of the main chain (Kurth et al., 2002). The carboxyl functionalized polymers were soluble in a variety of organic solvents, including acetone, methanol, and even in a combination of acetone/water (85/15, v/v). Following that, nanoparticles with diameters about 200 nm and a very narrow size distribution were produced. PHOD was grafted with PEG or polylactic acid (PLA), resulting in nanoparticles with even smaller diameter distributions (Renard et al., 2003). The carboxylated PHA (25% carboxyl side-groups) was utilized to produce a PEG grafted copolymer (Domenek et al., 2007), which enhanced the polymer’s solubility in polar solvents. The obtained polymer was soluble in a combination of acetone–water (30/70, v/v) and even partly in water when monomethoxy PEG with a molecular weight of 2000 g/mol was employed. Furthermore, aiming to add a functional carboxyl group on the PHA, PHOU was oxidized with OsO_4_ in DMF in the presence of Oxone (Stigers and Tew, 2003). When compared to the KMnO_4_ procedure, the latter gave somewhat greater decrease in molecular weight; nevertheless, the resulting polyesters have comparable solubility.

The PHOU hydroxylation processes gave hydroxyl-functional PHA. When PHOU interacted with 9-borobicyclononane (9-BBN) (Eroğlu et al., 2005) or with a BH3–THF complex (Renard et al., 2005), the hydroxylation was quantitative. After converting the vinyl groups to hydroxyl groups, the resulting poly (3-hydroxyundec-10-enoate) (PHU) with 100% unsaturated repeating units was almost completely soluble in water (Eroğlu et al., 2005), whereas the hydroxylated PHOU (obtained from a copolymer with 25% unsaturated side-groups) showed a solubility behavior that was similar to 25% PHOD-COOH (Kurth et al., 2002). The application of thiolene click chemistry, in which PHAs with unsaturated side chains react with the appropriate thiol derivative, is another approach to carboxylate or hydroxylate PHAs. For example, thiolene click chemistry was employed in two stages to produce a comb-like hydroxyl functionalized polymer (Hany et al., 2004). 1) The side-groups were extended using tridecylamine or 1-octadecanol, and the resulting product had a higher molecular weight due to the longer side chains. 2) In presence of Azobisisobutyronitrile (AIBN), PHOU with 25% unsaturated side groups was reacted with 11-mercaptoundecanoic acid. Such comb-like copolymer thermally degraded in two stages: 1) The polyester chain deteriorated at 300°C. 2) The following phase, which occurred between 300 and 400°C, was linked with the degradation of polyethylene-like side chains, confirming the hypothesized copolymer structure. Another fascinating example of the thiolene click chemistry is the production of amphiphilic PHA when soybean oil PHOU (PHOSy) was reacted with 3-thioglycerol or 3-mercaptopropionic acid under UV light (Hazer, 2015). Poly (3-hydroxy octanoate-*co*-soybean oil polymer) (PHOSy) is a polyester produced by *P. oleovorans* using soybean oil as the sole carbon source. From a starting point of 90°, the hydrophilic functional groups reduced the water contact angle to around 60°. Similarly, hydroxyl- and carboxyl-functionalized PHAs (functionalization with 2-mercaptoethanol or 3-mercaptopropanoic acid) were synthesized from copolymers containing 3-hydroxybutyrate (HB) and 8, 36 or 65 mol% 3-hydroxy-10-undecenoate (HU) units (PHBU). The water contact angle of these polymers decreased in a similar manner. Following crosslinking of PHBU with pentaerythritol tetrakis (3-mercaptopropionate), the elongation to break of the sample increased from 90% (non-crosslinked) to 500% (Levine et al., 2016).

Furthermore, chlorination of the unsaturated units (olefinic bonds of unsaturated PHA), was demonstrated to be very effective and converted the soft and elastic PHA containing 10–55 mol% unsaturated repeating units to a crystalline material. Unfortunately, degradation occurred during the addition of chlorine, and the more chlorine was added, the higher the ratio of degradation was. For example, following synthesis of PHA containing 54 wt% chlorine, the polyester molecular weight decreased from 50,000 to 13,000 g/mol. In addition, when the chlorine content of the PHA increased, the *T*_*g*_ climbed from 50°C to 58°C (Arkin et al., 2000). Nevertheless, a novel polymer resulted from this postsynthetic modification, which might have some useful applications (Table 1).

**TABLE 1:** Several enzymatic and chemical modification of various PHAs and their applications.

	Produced polymer	Application	References
Enzymatic modification	Poly (10-O-3-hydroxyacyl-sucrose)	Biomedical applications	Gumel et al., (2013a)
	(R)-3-hydroxy-3-phenylpropionic acids and (R)-3-hydroxy-5-phenylpentanoic	Antimicrobial biomedical application	Snoch et al., (2019)
	(R)-3-hydroxyheptanoic acids and (R)-3-hydroxynonanoic		
	P (3HB-*co*-3HV)-ascorbic acid	Antioxidant active biomaterial	Bhatia et al., (2019)
Chemical modification	poly (3-hydroxy-5-(2,4-difluorophenoxy) pentanoate)	Scaffold for tissue engineering	Takagi et al., (2004)
	poly (3-hydroxyoctanoate-*co*-9-carboxy-3-hydroxydecanoate)	Biomedical applications	Renard et al., (2007)
	Epoxidized-PHOU	Biodegradable elastomers, hydrogels, and adhesives	Park et al., (1998a)
	dihydroxy-terminated PHO	Functionalizable nanoparticles and drug delivery systems	Andrade et al., (2002)

## Conclusions and Perspectives

The current interest in neat polymer modification and functionalization *via* chemical and enzymatic processes stems from the demand for biodegradable and biocompatible PHAs with novel or tailormade physical and thermal properties. The need for enhanced flexibility to incorporate specific traits that extend their niche applications, as well as to overcome the difficulties encountered during the *in vivo* production through conventional biosynthesis is also highly demanded. The effectiveness of these procedures is primarily determined by how well the alteration imparts the intended trait(s) to the polymer, allowing it to execute the specified functions effectively. Optimal modification requires intelligent adjustment of process parameters such as reactant or sample concentration, treatment dosage, catalyst loading, exposure time and others, depending on the kind of PHA to be changed and its intended applications. A highly hydrophobic and crystalline PHA may be changed into a unique polymer with desirable bioactive qualities, a significant improvement in elasticity, wettability and storage modulus characteristics, allowing the functionalized polymers also to be utilized in other new applications.

Many modifications of polymers can be made. These modifications can be done in the backbone of the polymer or in the side chains. In addition, even several modifications of the polymer can be made by adding one or more functional groups, like also more than one active group can be modified in the polymer structure. All these modifications may result in a significantly larger number of new polymers. Many of them may have unique characteristics from their unmodified predecessors, which may increase their use in many new biological applications.

## References

[B1] Abd El-malek F. Khairy H. Farag A. Omar S. (2020). The Sustainability of Microbial Bioplastics, Production and Applications. Int. J. Biol. Macromolecules 157, 319–328. 10.1016/j.ijbiomac.2020.04.076 32315677

[B2] Abd El-malek F. Rofeal M. Farag A. Omar S. Khairy H. (2021). Polyhydroxyalkanoate Nanoparticles Produced by marine Bacteria Cultivated on Cost Effective Mediterranean Algal Hydrolysate media. J. Biotechnol. 328, 95–105. 10.1016/j.jbiotec.2021.01.008 33485864

[B3] Andrade A. P. Witholt B. Hany R. Egli T. Li Z. (2002). Preparation and Characterization of Enantiomerically Pure Telechelic Diols from mcl−Poly[(R)-3-hydroxyalkanoates]. Macromolecules 35, 684–689. 10.1021/ma011420r

[B4] Anis S. N. S. Mohd Annuar M. S. Simarani K. (2018). Microbial Biosynthesis Andin Vivodepolymerization of Intracellular Medium‐chain‐length Poly‐3‐hydroxyalkanoates as Potential Route to Platform Chemicals. Biotechnol. Appl. Biochem. 65, 784–796. 10.1002/bab.1666 29806235

[B5] Ansari N. F. Amirul A. A. (2013). Preparation and Characterization of Polyhydroxyalkanoates Macroporous Scaffold through Enzyme-Mediated Modifications. Appl. Biochem. Biotechnol. 170, 690–709. 10.1007/s12010-013-0216-0 23604967

[B6] Arkin A. H. Hazer B. Borcakli M. (2000). Chlorination of Poly(3-Hydroxy Alkanoates) Containing Unsaturated Side Chains. Macromolecules 33, 3219–3223. 10.1021/ma991535j

[B7] Arkin A. H. Hazer B. (2002). Chemical Modification of Chlorinated Microbial Polyesters. Biomacromolecules 3, 1327–1335. 10.1021/bm020079v 12425672

[B8] Arslan H. Hazer B. Yoon S. C. (2007). Grafting of Poly(3-Hydroxyalkanoate) and Linoleic Acid onto Chitosan. J. Appl. Polym. Sci. 103, 81–89. 10.1002/app.24276

[B9] Ashby R. D. Foglia T. A. Solaiman D. K. Y. Liu C.-K. Nuñez A. Eggink G. (2000). Viscoelastic Properties of Linseed Oil-Based Medium Chain Length Poly(hydroxyalkanoate) Films: Effects of Epoxidation and Curing. Int. J. Biol. Macromolecules 27, 355–361. 10.1016/S0141-8130(00)00140-9 10998494

[B10] Babinot J. Renard E. Langlois V. (2011). Controlled Synthesis of Well Defined Poly(3-Hydroxyalkanoate)s-Based Amphiphilic Diblock Copolymers Using Click Chemistry. Macromol. Chem. Phys. 212, 278–285. 10.1002/macp.201000562

[B11] Bassas-Galià M. Gonzalez A. Micaux F. Gaillard V. Piantini U. Schintke S. (2015). Chemical Modification of Polyhydroxyalkanoates (PHAs) for the Preparation of Hybrid Biomaterials. Chimia 69, 627–630. 10.2533/chimia.2015.627 26598409

[B12] Bear M.-M. Leboucher-Durand M.-A. Langlois V. Lenz R. W. Goodwin S. Guérin P. (1997). Bacterial Poly-3-Hydroxyalkenoates with Epoxy Groups in the Side Chains. Reactive Funct. Polym. 34, 65–77. 10.1016/S1381-5148(97)00024-2

[B13] Bhatia S. K. Wadhwa P. Hong J. W. Hong Y. G. Jeon J.-M. Lee E. S. (2019). Lipase Mediated Functionalization of Poly(3-Hydroxybutyrate-Co-3-Hydroxyvalerate) with Ascorbic Acid into an Antioxidant Active Biomaterial. Int. J. Biol. Macromolecules 123, 117–123.‏ 10.1016/j.ijbiomac.2018.11.052 30428310

[B14] Chen C. Fei B. Peng S. Wu H. Zhuang Y. Chen X. (2002). Synthesis and Characterization of Poly(β-Hydroxybutyrate) and Poly(ε-Caprolactone) Copolyester by Transesterification. J. Polym. Sci. B Polym. Phys. 40, 1893–1903. 10.1002/polb.10242

[B15] Chen G.-Q. Wu Q. (2005). Microbial Production and Applications of Chiral Hydroxyalkanoates. Appl. Microbiol. Biotechnol. 67, 592–599. 10.1007/s00253-005-1917-2 15700123

[B16] Chen X. Martin B. D. Neubauer T. K. Linhardt R. J. Dordick J. S. Rethwisch D. G. (1995). Enzymatic and Chemoenzymatic Approaches to Synthesis of Sugar-Based Polymer and Hydrogels. Carbohydr. Polym. 28, 15–21. 10.1016/0144-8617(95)00082-8

[B17] Chen Z. Cheng S. Li Z. Xu K. Chen G.-Q. (2009a). Synthesis, Characterization and Cell Compatibility of Novel Poly(ester Urethane)s Based on Poly(3-Hydroxybutyrate-Co-4-Hydroxybutyrate) and Poly(3-Hydroxybutyrate-Co-3-Hydroxyhexanoate) Prepared by Melting Polymerization. J. Biomater. Sci. Polym. Edition 20, 1451–1471. 10.1163/092050609X12457419007621 19622282

[B18] Chen Z. Cheng S. Xu K. (2009b). Block Poly(ester-Urethane)s Based on Poly(3-Hydroxybutyrate-Co-4-Hydroxybutyrate) and Poly(3-Hydroxyhexanoate-Co-3-Hydroxyoctanoate). Biomaterials 30, 2219–2230. 10.1016/j.biomaterials.2008.12.078 19167751

[B19] Ch’ng D. H.-E. Sudesh K. (2013). Densitometry Based Microassay for the Determination of Lipase Depolymerizing Activity on Polyhydroxyalkanoate. AMB Express 3, 1–1122. 10.1186/2191-0855-3 23657221 PMC3671206

[B20] Chung A.-L. Jin H.-L. Huang L.-J. Ye H.-M. Chen J.-C. Wu Q. (2011). Biosynthesis and Characterization of Poly(3-Hydroxydodecanoate) by β-Oxidation Inhibited Mutant of Pseudomonas Entomophila L48. Biomacromolecules 12, 3559–3566. 10.1021/bm200770m 21838281

[B21] Córdova A. (2001). Synthesis of Amphiphilic Poly(ε-Caprolactone) Macromonomers by Lipase Catalysis. Biomacromolecules 2, 1347–1351. 10.1021/bm0101015 11777414

[B22] Dai S. Li Z. (2008). Enzymatic Preparation of Novel Thermoplastic Di-block Copolyesters Containing Poly[(R)-3-hydroxybutyrate] and Poly(ϵ-Caprolactone) Blocks via Ring-Opening Polymerization. Biomacromolecules 9, 1883–1893. 10.1021/bm8001396 18540675

[B23] Domenek S. Langlois V. Renard E. (2007). Bacterial Polyesters Grafted with Poly(ethylene Glycol): Behaviour in Aqueous media. Polym. Degrad. Stab. 92, 1384–1392. 10.1016/j.polymdegradstab.2007.03.001

[B24] El-Malek F. A. Rofeal M. Zabed H. M. Nizami A.-S. Rehan M. Qi X. (2021). Microorganism-mediated Algal Biomass Processing for Clean Products Manufacturing: Current Status, Challenges and Future Outlook. Fuel 1, 122612.‏ 10.1016/j.fuel.2021.122612

[B25] El-Malek F. A. Farag A. Omar S. Khairy H. (2020). Polyhydroxyalkanoates (PHA) from Halomonas pacifica ASL10 and Halomonas Salifodiane ASL11 Isolated from Mariout Salt Lakes. Int. J. Biol. Macromolecules 161, 1318–1328. 10.1016/j.ijbiomac.2020.07.258 32755698

[B26] Eroğlu M. S. Hazer B. Ozturk T. Caykara T. (2005). Hydroxylation of Pendant Vinyl Groups of Poly(3-Hydroxy Undec-10-Enoate) in High Yield. J. Appl. Polym. Sci. 97, 2132–2139. 10.1002/app.21943

[B27] Erol A. Rosberg D. B. H. Hazer B. Göncü B. S. (2020). Biodegradable and Biocompatible Radiopaque Iodinated Poly-3-Hydroxy Butyrate: Synthesis, Characterization and *In Vitro*/*In Vivo* X-ray Visibility. Polym. Bull. 77 (1), 275–289. 10.1007/s00289-019-02747-6

[B28] Gan Z. Liang Q. Zhang J. Jing X. (1997). Enzymatic Degradation of Poly(ε-Caprolactone) Film in Phosphate Buffer Solution Containing Lipases. Polym. Degrad. Stab. 56, 209–213. 10.1016/S0141-3910(96)00208-X

[B29] Gangoiti J. Santos M. Llama M. J. Serra J. L. (2010). Production of Chiral ( R )-3-Hydroxyoctanoic Acid Monomers, Catalyzed by Pseudomonas Fluorescens GK13 Poly(3-Hydroxyoctanoic Acid) Depolymerase. Appl. Environ. Microbiol. 76, 3554–3560. 10.1128/AEM.00337-10 20400568 PMC2876462

[B30] Gangoiti J. Santos M. Prieto M. A. de la Mata I. Serra J. L. Llama M. J. (2012). Characterization of a Novel Subgroup of Extracellular Medium-Chain-Length Polyhydroxyalkanoate Depolymerases from Actinobacteria. Appl. Environ. Microbiol. 78, 7229–7237. 10.1128/AEM.01707-12 22865072 PMC3457088

[B31] González-Miró M. Radecker A.-M. Rodríguez-Noda L. M. Fariñas-Medina M. Zayas-Vignier C. Hernández-Cedeño M. (2018). Design and Biological Assembly of Polyester Beads Displaying Pneumococcal Antigens as Particulate Vaccine. ACS Biomater. Sci. Eng. 4, 3413–3424. 10.1021/acsbiomaterials.8b00579 33435075

[B32] Gumel A. Aris M. Annuar M. (2015). Modification of Polyhydroxyalkanoates (PHAs). RSC Green. Chem. 2015, 141–182.

[B33] Gumel A. M. Annuar M. S. M. Chisti Y. Heidelberg T. (2012a). Ultrasound Assisted Lipase Catalyzed Synthesis of Poly-6-Hydroxyhexanoate. Ultrason. Sonochem. 19, 659–667. 10.1016/j.ultsonch.2011.10.016 22105013

[B34] Gumel A. M. Annuar M. S. M. Heidelberg T. (2012b). Biosynthesis and Characterization of Polyhydroxyalkanoates Copolymers Produced by Pseudomonas Putida Bet001 Isolated from palm Oil Mill Effluent. PLOS ONE 7, e45214. 10.1371/journal.pone.0045214 23028854 PMC3447943

[B35] Gumel A. M. Annuar M. S. M. Heidelberg T. Chisti Y. (2011). Thermo-kinetics of Lipase-Catalyzed Synthesis of 6-O-Glucosyldecanoate. Bioresour. Tech. 102, 8727–8732. 10.1016/j.biortech.2011.07.024 21816608

[B36] Gumel A. M. Annuar M. S. M. Heidelberg T. (2013a). Enzymatic Synthesis of 6-O-Glucosyl-Poly(3-Hydroxyalkanoate) in Organic Solvents and Their Binary Mixture. Int. J. Biol. Macromolecules 55, 127–136. 10.1016/j.ijbiomac.2012.12.028 23305702

[B37] Gumel A. M. Annuar S. M. Heidelberg T. (2013b). Single-step Lipase-Catalyzed Functionalization of Medium-Chain-Length Polyhydroxyalkanoates. J. Chem. Technol. Biotechnol. 88, 1328–1335. 10.1002/jctb.3980

[B38] Ha S. H. Hiep N. M. Lee S. H. Koo Y.-M. (2010). Optimization of Lipase-Catalyzed Glucose Ester Synthesis in Ionic Liquids. Bioproc. Biosyst Eng 33, 63–70. 10.1007/s00449-009-0363-4 19680693

[B39] Hany R. Böhlen C. Geiger T. Hartmann R. Kawada J. Schmid M. (2004). Chemical Synthesis of Crystalline Comb Polymers from Olefinic Medium-Chain-Length Poly[3-Hydroxyalkanoates]. Macromolecules 37, 385–389. 10.1021/ma0304426

[B40] Haraźna K. Cichoń E. Skibiński S. Witko T. Solarz D. Kwiecień I. (2020). Physicochemical and Biological Characterisation of Diclofenac Oligomeric Poly(3-Hydroxyoctanoate) Hybrids as β-TCP Ceramics Modifiers for Bone Tissue Regeneration. Ijms 21, 249452. 10.3390/ijms21249452 PMC7763618 33322564

[B41] Hazer B. (2015). Simple Synthesis of Amphiphilic Poly(3-Hydroxy Alkanoate)s with Pendant Hydroxyl and Carboxylic Groups via Thiol-Ene Photo Click Reactions. Polym. Degrad. Stab. 119, 159–166. 10.1016/j.polymdegradstab.2015.04.024

[B42] Hiroe A. Sakurai T. Mizuno S. Miyahara Y. Goto S. Yamada M. (2021). Microbial Oversecretion of (R)-3-hydroxybutyrate Oligomer with Diethylene Glycol Terminal as a Macromonomer for Polyurethane Synthesis. Int. J. Biol. Macromolecules 167, 1290–1296. ‏. 10.1016/j.ijbiomac.2020.11.083 33202278

[B43] Ihssen J. Magnani D. Thöny-Meyer L. Ren Q. (2009). Use of Extracellular Medium Chain Length Polyhydroxyalkanoate Depolymerase for Targeted Binding of Proteins to Artificial Poly[(3-Hydroxyoctanoate)-Co-(3-Hydroxyhexanoate)] Granules. Biomacromolecules 10, 1854–1864. 10.1021/bm9002859 19459673

[B44] Imamura T. Kenmoku T. Honma T. Kobayashi S. Yano T. (2001). Direct Biosynthesis of Poly(3-Hydroxyalkanoates) Bearing Epoxide Groups. Int. J. Biol. Macromolecules 29, 295–301. 10.1016/S0141-8130(01)00179-9 11718827

[B45] Ito Y. Inoue M. Liu S. Q. Imanishi Y. (1993). Cell Growth on Immobilized Cell Growth Factor. 6. Enhancement of Fibroblast Cell Growth by Immobilized Insulin And/or Fibronectin. J. Biomed. Mater. Res. 27, 901–907. 10.1002/jbm.820270709 8360217

[B46] Jaeger K. E. Steinbüchel A. Jendrossek D. (1995). Substrate Specificities of Bacterial Polyhydroxyalkanoate Depolymerases and Lipases: Bacterial Lipases Hydrolyze poly(omega-hydroxyalkanoates). Appl. Environ. Microbiol. 61, 3113–3118. 10.1128/aem.61.8.3113-3118.1995 7487042 PMC167586

[B47] Jeepery I. F. Sudesh K. Abe H. (2021). Miscibility and Enzymatic Degradability of Poly(3-Hydroxybutyrate-Co-3-Hydroxyhexanoate)-Based Polyester Blends by PHB Depolymerase and Lipase. Polym. Degrad. Stab. 192, 109692. 10.1016/j.polymdegradstab.2021.109692

[B48] Jendrossek D. Handrick R. (2002). Microbial Degradation of Polyhydroxyalkanoates. Annu. Rev. Microbiol. 56, 403–432. 10.1146/annurev.micro.56.012302.160838 12213937

[B49] Kai D. Loh X. J. (2014). Polyhydroxyalkanoates: Chemical Modifications toward Biomedical Applications. ACS Sust. Chem. Eng. 2, 106–119. 10.1021/sc400340p

[B50] Kamachi M. Zhang S. Goodwin S. Lenz R. W. (2001). Enzymatic Polymerization and Characterization of New Poly(3-Hydroxyalkanoate)s by a Bacterial Polymerase. Macromolecules 34, 6889–6894. 10.1021/ma010081z

[B51] Kamal M. Z. Ahmad S. Molugu T. R. Vijayalakshmi A. Deshmukh M. V. Sankaranarayanan R. (2011). *In Vitro* evolved Non-aggregating and Thermostable Lipase: Structural and Thermodynamic Investigation. J. Mol. Biol. 413, 726–741. 10.1016/j.jmb.2011.09.002 21925508

[B52] Kamal M. Z. Yedavalli P. Deshmukh M. V. Rao N. M. (2013). Lipase in Aqueous-Polar Organic Solvents: Activity, Structure, and Stability. Protein Sci. 22, 904–915. 10.1002/pro.2271 23625694 PMC3719085

[B53] Kawata T. Ogino H. (2009). Enhancement of the Organic Solvent-Stability of the LST-03 Lipase by Directed Evolution. Biotechnol. Prog. 25, NA. 10.1002/btpr.264 19731302

[B54] Kawata Y. Ando H. Matsushita I. Tsubota J. (2014). Efficient Secretion of (R)-3-hydroxybutyric Acid from Halomonas Sp. KM-1 by Nitrate Fed-Batch Cultivation with Glucose under Microaerobic Conditions. Bioresour. Tech. 156, 400–403. 10.1016/j.biortech.2014.01.073 24503050

[B55] Kessler F. Marconatto L. Rodrigues R. D. S. B. Lando G. A. Schrank A. Vainstein M. H. (2014). Biodegradation Improvement of Poly(3-Hydroxy-Butyrate) Films by Entomopathogenic Fungi and UV-Assisted Surface Functionalization. J. Photochem. Photobiol. B: Biol. 130, 57–67. 10.1016/j.jphotobiol.2013.11.002 24300992

[B56] Kim H.-N. Lee J. Kim H.-Y. Kim Y.-R. (2009). Enzymatic Synthesis of a Drug Delivery System Based on Polyhydroxyalkanoate-Protein Block Copolymers. Chem. Commun. 46, 7104–7106. 10.1039/B912871A 19920997

[B57] Kim H. Y. Ryu J. H. Chu C. W. Son G. M. Jeong Y.-I. Kwak T.-W. (2014). Paclitaxel-incorporated Nanoparticles Using Block Copolymers Composed of Poly(ethylene Glycol)/poly(3-Hydroxyoctanoate). Nanoscale Res. Lett. 9, 1–10. 10.1186/1556-276X-9-525 25288916 PMC4184469

[B58] Kim Y. J. Kang I.-K. Huh M. W. Yoon S.-C. (2000). Surface Characterization and *In Vitro* Blood Compatibility of Poly(ethylene Terephthalate) Immobilized with Insulin And/or Heparin Using Plasma Glow Discharge. Biomaterials 21, 121–130. 10.1016/S0142-9612(99)00137-4 10632394

[B59] Klibanov A. M. (2001). Improving Enzymes by Using Them in Organic Solvents. Nature 409, 241–246. 10.1038/35051719 11196652

[B60] Knoll M. Hamm T. M. Wagner F. Martinez V. Pleiss J. (2009). The PHA Depolymerase Engineering Database: A Systematic Analysis Tool for the Diverse Family of Polyhydroxyalkanoate (PHA) Depolymerases. BMC Bioinformatics 10, 89. 10.1186/1471-2105-10-89 19296857 PMC2666664

[B61] Kunasundari B. Sudesh K. (2011). Isolation and Recovery of Microbial Polyhydroxyalkanoates. EXPRESS Polym. Lett. 5, 620–634. 10.3144/expresspolymlett.2011.60

[B62] Kurth N. Renard E. Brachet F. Robic D. Guerin P. Bourbouze R. (2002). Poly(3-hydroxyoctanoate) Containing Pendant Carboxylic Groups for the Preparation of Nanoparticles Aimed at Drug Transport and Release. Polymer 43, 1095–1101. 10.1016/S0032-3861(01)00692-9

[B63] Kwiecień M. Adamus G. Kowalczuk M. (2013). Selective Reduction of PHA Biopolyesters and Their Synthetic Analogues to Corresponding PHA Oligodiols Proved by Structural Studies. Biomacromolecules 14, 1181–1188. 10.1021/bm400141s 23464789

[B64] Kwon O. H. Lee I. S. Ko Y.-G. Meng W. Jung K.-H. Kang I.-K. (2007). Electrospinning of Microbial Polyester for Cell Culture. Biomed. Mater. 2, S52–S58. 10.1088/1748-6041/2/1/S08 18458420

[B65] Lee J. Jung S.-G. Park C.-S. Kim H.-Y. Batt C. A. Kim Y.-R. (2011). Tumor-specific Hybrid Polyhydroxybutyrate Nanoparticle: Surface Modification of Nanoparticle by Enzymatically Synthesized Functional Block Copolymer. Bioorg. Med. Chem. Lett. 21, 2941–2944. 10.1016/j.bmcl.2011.03.058 21489794

[B66] Lee M. Y. Cha S. Y. Park W. H. (1999a). Crosslinking of Microbial Copolyesters with Pendant Epoxide Groups by Diamine. Polymer 40, 3787–3793. 10.1016/S0032-3861(98)00612-0

[B67] Lee M. Y. Park W. H. (1999). Epoxidation of Bacterial Polyesters with Unsaturated Side Chains V. Effect of Crosslinking on thermal Degradation of Epoxidized Polymers. Polym. Degrad. Stab. 65, 137–142. 10.1016/S0141-3910(98)00229-8

[B68] Lee M. Y. Park W. H. (2000). Preparation of Bacterial Copolyesters with Improved Hydrophilicity by Carboxylation. Macromol. Chem. Phys. 201, 2771–2774. 10.1002/1521-3935(20001201)201:18<2771:aid-macp2771>3.0.co;2-v

[B69] Lee S. Y. Lee Y. Wang F. (1999b). Chiral Compounds from Bacterial Polyesters: Sugars to Plastics to fine Chemicals. Biotechnol. Bioeng. 65, 363–368. 10.1002/(sici)1097-0290(19991105)65:3<363:aid-bit15>3.0.co;2-1 10486136

[B70] Levine A. C. Heberlig G. W. Nomura C. T. (2016). Use of Thiol-Ene Click Chemistry to Modify Mechanical and thermal Properties of Polyhydroxyalkanoates (PHAs). Int. J. Biol. Macromolecules 83, 358–365. 10.1016/j.ijbiomac.2015.11.048 26616449

[B71] Levine A. C. Sparano A. Twigg F. F. Numata K. Nomura C. T. (2015). Influence of Cross-Linking on the Physical Properties and Cytotoxicity of Polyhydroxyalkanoate (PHA) Scaffolds for Tissue Engineering. ACS Biomater. Sci. Eng. 1 (7), 567–576. 10.1021/acsbiomaterials.5b00052 33434973

[B72] Liu S. Q. Ito Y. Imanishi Y. (1993). Cell Growth on Immobilized Cell Growth Factor. 9. Covalent Immobilization of Insulin, Transferrin, and Collagen to Enhance Growth of Bovine Endothelial Cells. J. Biomed. Mater. Res. 27, 909–915. 10.1002/jbm.820270710 8360218

[B73] Lu X.-Y. Li M.-C. Zhu X.-L. Fan F. Wang L.-L. Ma J.-G. (2014). Microbial Synthesized Biodegradable PHBHHxPEG Hybrid Copolymer as an Efficient Intracellular Delivery Nanocarrier for Kinase Inhibitor. BMC Biotechnol. 14, 4. 10.1186/1472-6750-14-4 24438107 PMC3909372

[B74] Martin B. D. Ampofo S. A. Linhardt R. J. Dordick J. S. (1992). Biocatalytic Synthesis of Sugar-Containing Polyacrylate-Based Hydrogels. Macromolecules 25, 7081–7085. 10.1021/ma00052a001

[B75] Michalak M. Kwiecien I. Kwiecien M. Adamus G. Odelius K. Hakkarainen M. (2017). Diversifying Polyhydroxyalkanoates - End-Group and Side-Chain Functionality. Cos 14, 757–767. 10.2174/1570179414666161115150146

[B76] Mishra M. K. Kumaraguru T. Sheelu G. Fadnavis N. W. (2009). Lipase Activity of Lecitase Ultra: Characterization and Applications in Enantioselective Reactions. Tetrahedron: Asymmetry 20, 2854–2860. 10.1016/j.tetasy.2009.11.012

[B77] Mukai K. Doi Y. Sema Y. Tomita K. (1993). Substrate Specificities in Hydrolysis of Polyhydroxyalkanoates by Microbial Esterases. Biotechnol. Lett. 15, 601–604. 10.1007/BF00138548

[B78] Müller H.-M. Seebach D. (1993). Poly(hydroxyalkanoates): A Fifth Class of Physiologically Important Organic Biopolymers? Angew. Chem. Int. Ed. Engl. 32, 477–502. 10.1002/anie.199304771

[B79] Nguyen S. Marchessault R. H. (2004). Synthesis and Properties of Graft Copolymers Based on Poly(3-Hydroxybutyrate) Macromonomers. Macromol. Biosci. 4, 262–268. 10.1002/mabi.200300088 15468216

[B80] Ogino H. Ishikawa H. (2001). Enzymes Which Are Stable in the Presence of Organic Solvents. J. Biosci. Bioeng. 91, 109–116. 10.1016/S1389-1723(01)80051-7 16232960

[B81] Paik H.-J. Kim Y.-R. Orth R. N. Ober C. K. Coates G. W. Batt C. A. (2005). End-functionalization of Poly(3-Hydroxybutyrate)via Genetic Engineering for Solid Surface Modification. Chem. Commun. 15, 1956–1958. 10.1039/B415809A 15834470

[B82] Park W. H. Lenz R. W. Goodwin S. (1999). Epoxidation of Bacterial Polyesters with Unsaturated Side Chains: IV. Thermal Degradation of Initial and Epoxidized Polymers. Polym. Degrad. Stab. 63, 287–291. 10.1016/S0141-3910(98)00107-4

[B83] Park W. H. Lenz R. W. Goodwin S. (1998b). Epoxidation of Bacterial Polyesters with Unsaturated Side Chains. III. Crosslinking of Epoxidized Polymers. J. Polym. Sci. A. Polym. Chem. 36, 2389–2396. 10.1002/(SICI)1099-0518(19980930)36:13<2389:AID-POLA26>3.0.CO;2-5

[B84] Park W. H. Lenz R. W. Goodwin S. (1998a). Epoxidation of Bacterial Polyesters with Unsaturated Side Chains. II. Rate of Epoxidation and Polymer Properties. J. Polym. Sci. A. Polym. Chem. 36, 2381–2387. 10.1002/(sici)1099-0518(19980930)36:13<2381:aid-pola25>3.0.co;2-5

[B85] Pastorino L. Pioli F. Zilli M. Converti A. Nicolini C. (2004). Lipase-catalyzed Degradation of Poly(ε-Caprolactone). Enzyme Microb. Tech. 35, 321–326. 10.1016/j.enzmictec.2004.05.005

[B86] Ravenelle F. Marchessault R. H. (2002). One-Step Synthesis of Amphiphilic Diblock Copolymers from Bacterial Poly([R]-3-hydroxybutyric Acid). Biomacromolecules 3, 1057–1064. 10.1021/bm025553b 12217053

[B87] Reddy C. S. K. Ghai R. Rashmi V. C. Kalia V. C. (2003). Polyhydroxyalkanoates: an Overview. Bioresour. Tech. 87, 137–146. 10.1016/S0960-8524(02)00212-2 12765352

[B88] Rehm B. H. A. Antonio R. V. Spiekermann P. Amara A. A. Steinbüchel A. (2002). Molecular Characterization of the Poly(3-Hydroxybutyrate) (PHB) Synthase from Ralstonia Eutropha: *In Vitro* Evolution, Site-specific Mutagenesis and Development of a PHB Synthase Protein Model. Biochim. Biophys. Acta (Bba) - Protein Struct. Mol. Enzymol. 1594, 178–190. 10.1016/S0167-4838(01)00299-0 11825620

[B89] Rehm B. H. A. (2003). Polyester Synthases: Natural Catalysts for Plastics. Biochem. J. 376, 15–33. 10.1042/bj20031254 12954080 PMC1223765

[B90] Ren Q. Grubelnik A. Hoerler M. Ruth K. Hartmann R. Felber H. (2005). Bacterial Poly(hydroxyalkanoates) as a Source of Chiral Hydroxyalkanoic Acids. Biomacromolecules 6, 2290–2298. 10.1021/bm050187s 16004474

[B91] Renard E. Langlois V. Guérin P. (2007). Chemical Modifications of Bacterial Polyesters: from Stability to Controlled Degradation of Resulting Polymers. Corrosion Eng. Sci. Tech. 42, 300–311. 10.1179/174327807X238918

[B92] Renard E. Poux A. Timbart L. Langlois V. Guérin P. (2005). Preparation of a Novel Artificial Bacterial Polyester Modified with Pendant Hydroxyl Groups. Biomacromolecules 6, 891–896. 10.1021/bm049337+ 15762656

[B93] Renard E. Ternat C. Langlois V. Guerin P. (2003). Synthesis of Graft Bacterial Polyesters for Nanoparticles Preparation. Macromol. Biosci. 3, 248–252. 10.1002/mabi.200390033

[B94] Rofeal M. El-Malek F. A. Qi X. (2021). *In Vitro* assessment of green Polyhydroxybutyrate/chitosan Blend Loaded with Kaempferol Nanocrystals as a Potential Dressing for Infected Wounds. Nanotechnology 32, 375102. 10.1088/1361-6528/abf7ee 33853056

[B95] Santaniello E. Ferraboschi P. Grisenti P. (1993). Lipase-catalyzed Transesterification in Organic Solvents: Applications to the Preparation of Enantiomerically Pure Compounds. Enzyme Microb. Tech. 15, 367–382. 10.1016/0141-0229(93)90123-J

[B96] Sato S. Ono Y. Mochiyama Y. Sivaniah E. Kikkawa Y. Sudesh K. (2008). Polyhydroxyalkanoate Film Formation and Synthase Activity during *In Vitro* and *In Situ* Polymerization on Hydrophobic Surfaces. Biomacromolecules 9, 2811–2818. 10.1021/bm800566s 18771315

[B97] Shah D. T. Tran M. Berger P. A. Aggarwal P. Asrar J. Madden L. A. (2000). Synthesis and Properties of Hydroxy-Terminated Poly(hydroxyalkanoate)s. Macromolecules 33, 2875–2880. 10.1021/ma991773e

[B98] Shrivastav A. Kim H.-Y. Kim Y.-R. (20132013). Advances in the Applications of Polyhydroxyalkanoate Nanoparticles for Novel Drug Delivery System. Biomed. Res. Int. 2013, 1–12. 10.1155/2013/581684 PMC3741897 23984383

[B99] Snoch W. Stępień K. Prajsnar J. Staroń J. Szaleniec M. Guzik M. (2019). Influence of Chemical Modifications of Polyhydroxyalkanoate-Derived Fatty Acids on Their Antimicrobial Properties. Catalysts 9, 510. 10.3390/catal9060510

[B100] Sparks J. Scholz C. (2009). Evaluation of a Cationic Poly(β-Hydroxyalkanoate) as a Plasmid DNA Delivery System. Biomacromolecules 10, 1715–1719. 10.1021/bm900372x 19445454

[B101] Sparks J. Scholz C. (2008). Synthesis and Characterization of a Cationic Poly(β-Hydroxyalkanoate). Biomacromolecules 9, 2091–2096. 10.1021/bm8005616 18646823

[B102] SteinbÃ¼chel A. Valentin H. E. (1995). Diversity of Bacterial Polyhydroxyalkanoic Acids. FEMS Microbiol. Lett. 128, 219–228. 10.1111/j.1574-6968.1995.tb07528.x

[B103] Stevens M. P. (2009). Polymer Chemistry: An Introduction. USA: Oxford University Press.

[B104] Stigers D. J. Tew G. N. (2003). Poly(3-hydroxyalkanoate)s Functionalized with Carboxylic Acid Groups in the Side Chain. Biomacromolecules 4, 193–195. 10.1021/bm025728h 12625711

[B105] Taguchi S. Doi Y. (2004). Evolution of Polyhydroxyalkanoate(PHA) Production System by"Enzyme Evolution": Successful Case Studies of Directed Evolution. Macromol. Biosci. 4, 145–156. 10.1002/mabi.200300111 15468204

[B106] Takagi Y. Yasuda R. Maehara A. Yamane T. (2004). Microbial Synthesis and Characterization of Polyhydroxyalkanoates with Fluorinated Phenoxy Side Groups from Pseudomonas Putida. Eur. Polym. J. 40, 1551–1557. 10.1016/j.eurpolymj.2004.01.030

[B107] Tezcaner A. Bugra K. Hasırcı V. (2003). Retinal Pigment Epithelium Cell Culture on Surface Modified Poly(hydroxybutyrate-Co-Hydroxyvalerate) Thin Films. Biomaterials 24, 4573–4583. 10.1016/S0142-9612(03)00302-8 12951000

[B108] Timbart L. Renard E. Tessier M. Langlois V. (2007). Monohydroxylated Poly(3-Hydroxyoctanoate) Oligomers and its Functionalized Derivatives Used as Macroinitiators in the Synthesis of Degradable Diblock Copolyesters. Biomacromolecules 8, 1255–1265. 10.1021/bm060981t 17338561

[B109] Tokiwa Y. Fan H. Hiraguri Y. Kurane R. Kitagawa M. Shibatani S. (2000). Biodegradation of a Sugar Branched Polymer Consisting of Sugar, Fatty Acid, and Poly(vinyl Alcohol). Macromolecules 33, 1636–1639. 10.1021/ma981451v

[B110] Urbanek A. K. Mirończuk A. M. García-Martín A. Saborido A. De La Mata I. Arroyo M. (2020). Biochemical Properties and Biotechnological Applications of Microbial Enzymes Involved in the Degradation of Polyester-type Plastics. Biochim. Biophys. Acta (Bba) - Proteins Proteomics 1868, 140315. 10.1016/j.bbapap.2019.140315 31740410

[B111] Ushimaru K. Mizuno S. Honya A. Abe H. Tsuge T. (2017). Real-time Observation of Enzymatic Polyhydroxyalkanoate Polymerization Using High-Speed Scanning Atomic Force Microscopy. ACS omega 2 (1), 181–185. 10.1021/acsomega.6b00355 30023512 PMC6044693

[B112] Valentin H. E. Berger P. A. Gruys K. J. Filomena De Andrade Rodrigues M. Steinbüchel A. Tran M. (1999). Biosynthesis and Characterization of Poly(3-Hydroxy-4-Pentenoic Acid). Macromolecules 32, 7389–7395. 10.1021/ma9905167

[B113] Vastano M. Pellis A. Immirzi B. Dal Poggetto G. Malinconico M. Sannia G. (2017). Enzymatic Production of Clickable and PEGylated Recombinant Polyhydroxyalkanoates. Green. Chem. 19 (22), 5494–5504. 10.1039/C7GC01872J

[B114] Wang Y.-W. Mo W. Yao H. Wu Q. Chen J. Chen G.-Q. (2004). Biodegradation Studies of Poly(3-Hydroxybutyrate-Co-3-Hydroxyhexanoate). Polym. Degrad. Stab. 85, 815–821. 10.1016/j.polymdegradstab.2004.02.010

[B115] Wang Y.-W. Wu Q. Chen G.-Q. (2003). Reduced Mouse Fibroblast Cell Growth by Increased Hydrophilicity of Microbial Polyhydroxyalkanoates via Hyaluronan Coating. Biomaterials 24, 4621–4629. 10.1016/S0142-9612(03)00356-9 12951005

[B116] Wu C.-S. (2013). Preparation, Characterization and Biodegradability of Crosslinked tea Plant-Fibre-Reinforced Polyhydroxyalkanoate Composites. Polym. Degrad. Stab. 98 (8), 1473–1480. 10.1016/j.polymdegradstab.2013.04.013

[B117] Xue D. Fan X. Zhang Z. Lv W. (2016). The Synthesis of Hydroxybutyrate-Based Block Polyurethane from Telechelic Diols with Robust thermal and Mechanical Properties. J. Chem. 2016, 1–10. 10.1155/2016/9635165

[B118] Yadav G. D. Devi K. M. (2004). Immobilized Lipase-Catalysed Esterification and Transesterification Reactions in Non-aqueous media for the Synthesis of Tetrahydrofurfuryl Butyrate: Comparison and Kinetic Modeling. Chem. Eng. Sci. 59, 373–383. 10.1016/j.ces.2003.09.034

[B119] Yang X. Zhao K. Chen G.-Q. (2002). Effect of Surface Treatment on the Biocompatibility of Microbial Polyhydroxyalkanoates. Biomaterials 23, 1391–1397. 10.1016/S0142-9612(01)00260-5 11804295

[B120] Zhang C. Zhao L. Dong Y. Zhang X. Lin J. Chen Z. (2010). Folate-mediated Poly(3-Hydroxybutyrate-Co-3-Hydroxyoctanoate) Nanoparticles for Targeting Drug Delivery. Eur. J. Pharmaceutics Biopharmaceutics 76, 10–16. 10.1016/j.ejpb.2010.05.005 20472060

[B121] Zhou L. Chen Z. Chi W. Yang X. Wang W. Zhang B. (2012). Mono-methoxy-poly(3-hydroxybutyrate-co-4-hydroxybutyrate)-graft-hyper-branched Polyethylenimine Copolymers for siRNA Delivery. Biomaterials 33, 2334–2344. 10.1016/j.biomaterials.2011.11.060 22154621

